# Cruzain Inhibitors for Chagas Disease: Anticorrelated Optimisation Landscapes and the Multiparametric Path to Clinical Candidates

**DOI:** 10.1111/cbdd.70340

**Published:** 2026-06-14

**Authors:** Caroline Rodrigues Chaves dos Reis, Hellen Valério Chaves Moura de Souza, Lidia Corrêa Parra, Guilber Valério Chaves Moura de Souza, Bruna Costa Zorzanelli, Nubia Boechat, Lucas Villas Bôas Hoelz, Tácio Vinício Amorim Fernandes

**Affiliations:** ^1^ Fundação Oswaldo Cruz, Fiocruz, Instituto de Tecnologia em Fármacos, Programa de Pós‐Graduação em Pesquisa Translacional em Fármacos e Medicamentos Rio de Janeiro RJ Brazil; ^2^ Fundação Oswaldo Cruz, Fiocruz, Instituto de Tecnologia em Fármacos, Laboratório de Síntese de Fármacos (LASFAR) Rio de Janeiro RJ Brazil; ^3^ Universidade Federal Fluminense (UFF), Campus Praia Vermelha Niterói RJ Brazil; ^4^ Universidade Estácio de Sá, Campus Niterói Niterói RJ Brazil; ^5^ Centro Universitário Serra dos Órgãos (UNIFESO) Teresópolis RJ Brazil; ^6^ Instituto Federal do Rio de Janeiro (IFRJ), Laboratório Computacional de Química Medicinal (LCQM) Pinheiral RJ Brazil

**Keywords:** Chagas disease, computational drug design, cruzain, cysteine protease inhibitors, molecular dynamics, multiparametric optimisation, QSAR, translational failure, *Trypanosoma cruzi*

## Abstract

Over three decades, cruzain, the principal cysteine protease of *Trypanosoma cruzi* and a validated drug target for Chagas disease, has accumulated 215 inhibitors with IC_50_ ≤ 1 μM, more than 30 crystal structures, and no clinical candidates. This review argues that the resulting translational paradox reflects not target invalidity, but a persistent strategic mismatch between how cruzain inhibitors have been optimised and what intracellular efficacy requires. Across peptidyl vinyl sulfones, non‐peptidic scaffolds, and thiosemicarbazones, enzymatic potency has been systematically prioritised over the coupled determinants of intracellular exposure: permeability, metabolic stability, and cathepsin selectivity. Each inhibitor class fails for a distinct proximal reason, and these failures converge on the same optimisation bias. Computational approaches (docking, molecular dynamics, free‐energy perturbation and machine‐learning QSAR) have been applied with increasing sophistication, but against affinity‐centred objectives that underrepresent exposure constraints. As its principal contribution, this work proposes a multiparametric optimisation framework, provisional viability benchmarks, and a feasibility‐envelope concept that reframes cruzain inhibitor discovery as a constraint‐satisfaction rather than a potency‐maximisation problem. The framework outlines provisional quantitative criteria that a next‐generation cruzain lead would likely need to satisfy and explains why structurally tractable intracellular cysteine protease targets can remain clinically unrealised.

## Graphical abstract

Three decades of cruzain‐targeted drug discovery have yielded 215 sub‐micromolar inhibitors but no clinical candidates. This review diagnoses a systemic translational failure driven by single‐parameter optimisation and proposes a multiparametric framework integrating enzymatic potency, cathepsin selectivity, intracellular exposure, and metabolic stability as coupled design constraints.

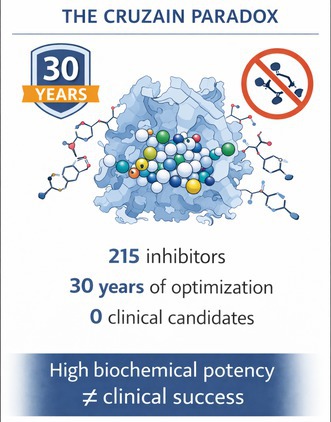

## 1 Introduction: The 30‐Year Paradox in Cruzain‐Targeted Drug Discovery

Few targets in Chagas disease (CD) drug discovery illustrate the gap between biological plausibility and translational success as clearly as cruzain (CZ), the principal cysteine protease of *Trypanosoma cruzi* ( *T. cruzi* ). Over three decades, CZ has accumulated the hallmarks of a high‐confidence preclinical target: absence of a direct human orthologue (Cazzulo et al. 2001), genetic essentiality across all parasite life‐cycle stages (Engel et al. 1998; Doyle et al. 2007), structural characterisation by more than 30 high‐resolution crystal structures (McGrath et al. 1995; Silva et al. 2019), and pharmacological proof‐of‐concept through K777, a subnanomolar inhibitor (*K*_*i*_ = 0.2 nM) that delivers parasitological cure in murine acute infection (Engel et al. 1998; Kerr et al. 2009; Doyle et al. 2007). We systematically curated the ChEMBL target CHEMBL3563 (cruzipain; European Bioinformatics Institute 2026, accessed February 2026) by applying successive pChEMBL thresholds (full workflow in Supporting Information). At pChEMBL ≥ 6 (IC_50_ ≤ 1 μM), 215 unique inhibitors qualified after deduplication by Molecule ChEMBL ID; raising the threshold to pChEMBL ≥ 7 (IC_50_ ≤ 100 nM) yielded 132 compounds (~60% of the potency‐qualified subset; see Figure S1). Yet not one compound has entered clinical development. This review argues that the obstacle lies not in target legitimacy but in a recurring optimisation mismatch: three decades of predominantly single‐parameter optimisation that prioritised enzymatic potency over the coupled constraints of permeability, metabolic stability, and cathepsin (CTS) selectivity, which often behave as competing optimisation demands—the parameters that ultimately govern intracellular drug exposure and clinical viability.

Retrospective analysis of the CZ inhibitor landscape reveals a convergent failure pattern across three chemically distinct scaffold classes (Caputto et al. 2012; Du et al. 2002; Engel et al. 1998; Ferreira et al. 2019; McKerrow et al. 2009). Peptidyl vinyl sulfones maximise target engagement but fail to translate beyond preclinical models due to exposure, safety, and selectivity liabilities (Engel et al. 1998; Doyle et al. 2011). Non‐peptide scaffolds partially satisfy Lipinski drug‐likeness criteria but exhibit substantial disconnects between enzymatic IC_50_ and cellular EC_50_, spanning approximately one to two orders of magnitude across scaffold classes. Benzimidazole derivatives exhibit enzymatic IC_50_ values of 5–50 μM, yet cellular activity remains weak (> 100 μM; an approximately 6‐ to 20‐fold enzymatic‐to‐cellular disconnect) (Ferreira et al. 2014; Ríos et al. 2013; Paes et al. 2025; Jasinski et al. 2024). Similarly, thiosemicarbazones with enzymatic IC_50_ values as low as 380 nM retain trypanocidal activity only in the low‐μM range, indicating an approximate one‐order‐of‐magnitude enzyme‐to‐cell disconnect that Du et al. (2002) attributed to rapid metabolic quenching rather than permeability failure. These discrepancies reflect inadequate penetration of the parasitophorous vacuole that houses intracellular *T. cruzi* amastigotes, combined with active efflux‐mediated clearance (Alvarez et al. 2012). Notably, optimised cyclic imides achieved favourable enzymatic‐to‐cellular translation (compound 59: enzymatic IC_50_ = 0.6 μM vs. cellular EC_50_ = 1.0 μM; ~1.7‐fold), suggesting that this permeability barrier may be surmountable through rational design (Ferreira et al. 2019). Natural product‐derived compounds (IC_50_ = 15–100 μM) contribute structural diversity but lack the intrinsic potency required for lead optimisation (Duschak and Couto 2009; Ferreira et al. 2010; Salas‐Sarduy et al. 2013). The unifying explanation across all three classes is sequential single‐parameter optimisation (SPO): each scaffold was driven towards one measurable property (potency, drug‐likeness, or structural novelty), while the interconnected requirements for intracellular drug exposure were systematically subordinated. The result is a property landscape in which improvement along one parameter often comes at the expense of another, a constraint that iterative single‐parameter medicinal chemistry has proven poorly suited to navigate.

Rather than cataloguing CZ inhibitors, this review analyses why anticorrelated optimisation landscapes have impeded clinical translation, evaluates whether computational methodologies can navigate these constraints, and proposes a multiparametric framework with quantitative viability criteria generalisable to CTS‐family targets across neglected tropical diseases and oncology.

The following sections establish this diagnostic framework (Sections 3 and 4) and formalise the MPO solution (Section 5).

## 2 Cruzain as a Drug Target: Exceptional Tractability and Inherent Selectivity Constraints

Cruzain (CZ) is functionally active across all major *T. cruzi* life‐cycle stages (epimastigotes, trypomastigotes, and intracellular amastigotes; Engel et al. 1998; McKerrow et al. 2009), but the therapeutically decisive stage is the intracellular amastigote, which drives disease persistence in chronic infection (Pérez‐Molina and Molina 2018; Rassi Jr et al. 2010; see Figure S2). This biological reality imposes the central pharmacological constraint on CZ inhibitor design: compounds must achieve and sustain inhibitory concentrations not merely in plasma but within the acidic lysosomal compartment of a parasite that itself resides within a host cell, a delivery challenge substantially more complex than that faced by extracellular targets.

The papain‐like (C1) fold organises a bilobal active site, comprising an α‐helical L‐domain and a β‐sheet‐rich R‐domain, around a conserved Cys25‐His162‐Asn182 catalytic triad. This nucleophilic geometry is activated under mildly acidic conditions (McGrath et al. 1995; Lecaille et al. 2002). More than 30 high‐resolution CZ crystal structures collectively define this binding architecture at sub‐2.0 Å resolution (Silva et al. 2019). The S2 subsite, which is unusually deep, hydrophobic, and tolerant of bulky P2 substituents, serves as the principal pharmacophore anchor for structure‐based design (Gillmor et al. 1997). Such structural coverage is exceptional among parasitic cysteine protease targets; comparative targets, including falcipain‐2 (*Plasmodium falciparum*) and TbCatB (*Trypanosoma brucei*), have fewer than 15 and 10 structurally characterised inhibitor complexes, respectively (Kerr et al. 2009; Sajid and McKerrow 2002).

The target's essentiality is supported by convergent evidence. Genetic disruption and functional attenuation of the cruzain gene array result in *T. cruzi* parasites with severe epimastigote growth defects, impaired differentiation, and reduced host‐cell invasion (Engel et al. 1998; McKerrow et al. 2009). Chemical validation further substantiates this essentiality. The peptidomimetic vinyl sulfone K777 irreversibly inactivates CZ with subnanomolar enzymatic potency and achieves robust parasitological cure in murine models of acute *T. cruzi* infection following oral administration (Doyle et al. 2007; Engel et al. 1998). The pharmacological proof‐of‐concept for CZ is among the most robust reported for parasitic protease targets.

CZ localisation varies by life‐cycle stage, with direct pharmacological consequences. The enzyme operates across a pH gradient spanning approximately 5.5 (lysosomal lumen) to 7.4 (host‐cell cytoplasmic interface), requiring inhibitors to maintain binding competence across > 2 pH units (Jasinski et al. 2024). Furthermore, compounds must traverse the parasitophorous vacuole membrane in addition to the host‐cell plasma membrane, a cumulative permeability demand that is poorly captured by standard Caco‐2 or PAMPA assays. This pH‐partitioning constraint introduces a design paradox: weakly basic compounds that accumulate in acidic lysosomes (a desirable property for target access) may simultaneously suffer reduced binding affinity due to protonation‐dependent changes in the catalytic triad geometry (see Section 4.2).

In addition to pharmacokinetic constraints, a fundamental challenge to selectivity arises from evolutionary conservation. CZ shares only partial overall sequence identity with human CTSs. For example, 42.2% with CTSL (Eakin et al. 1992) and 38% homology with CTSS (Brak et al. 2008) in commonly cited comparisons yet its active‐site region is substantially more conserved, with > 70% sequence identity reported for active‐site residues across human CTSs (Sartori et al. 2019). The catalytic machinery is correspondingly highly conserved at both structural and functional levels. In addition, the S2 pocket of CZ shares with CTSB the unusual ability to accommodate both hydrophobic and basic residues, reflecting a key common feature at the base of this specificity‐determining subsite (Durrant et al. 2010; Alves et al. 2001). The Cys25‐His162‐Asn182 triad and the Gln19‐mediated oxyanion hole are structurally superimposable across clan CA cysteine proteases (Turk et al. 2012). While this conservation confirms CZ as a mechanistically druggable target, it imposes a significant constraint: potency‐driven optimisation within the S2 and S3 subsites, the primary focus of inhibitor development, often results in off‐target CTS activity. The translational paradox for CZ inhibitors may stem less from target invalidity than from the difficulty of achieving meaningful selectivity (> 100‐fold over host CTSs) through active‐site binding alone. Binding to the conserved Cys25‐His162‐Asn182 triad appears to be predominantly enthalpy‐driven, reflecting extensive hydrogen bonding with residues structurally conserved across host CTSs, a thermodynamic signature characteristic of clan CA cysteine protease inhibitors (Turk et al. 2012; Lecaille et al. 2002). Selectivity must therefore be engineered through interactions with non‐conserved residues, primarily within the S2 and S3 subsites. This creates a structural paradox: the catalytic core is highly conserved. At the same time, only a small, structurally heterogeneous peripheral region is available for distinguishing between CZ and host CTSs (see Figure S3 for structural context). These peripheral regions also exhibit substantial variability across the CZ isoform family, further narrowing the effective selectivity window. The convergent lines of evidence establishing CZ as a tractable yet selectivity‐constrained drug target are summarised in Table 1.

**TABLE 1:** Evidence base supporting cruzain target validity across structure, essentiality and chemical proof‐of‐concept.

Evidence axis	Key observation	Translational implication	Primary source
Structural characterisation	High‐resolution crystal structures of mature CZ reveal a well‐defined papain‐like fold with a deep, druggable active‐site cleft	Enables structure‐based drug design and rational inhibitor optimisation	McGrath et al. (1995)
Active‐site architecture	Conserved Cys25‐His162‐Asn182 catalytic triad with geometry supporting nucleophilic catalysis near the enzyme's acidic pH optimum; protonation‐state changes outside this regime have direct implications for inhibitor modelling	Predictable covalent and non‐covalent inhibition mechanisms	Lecaille et al. (2002)
Structural tractability	More than 30 crystallographic structures of CZ‐ligand complexes are available	Unusually rich structural foundation for iterative design	Silva et al. (2019)
Genetic/functional essentiality	Disruption or functional attenuation of the cruzain gene arrays severely impairs parasite growth, differentiation, and host‐cell invasion	Establishes CZ as a non‐redundant enzyme across multiple life‐cycle stages	Engel et al. (1998)
Chemical validation	Pharmacological inhibition of CZ arrests parasite replication in vitro and in vivo	Confirms target engagement is sufficient for antiparasitic efficacy	Engel et al. 1998
In vivo proof‐of‐concept	Vinyl sulfone inhibitor K777 produces parasitological cure in murine acute infection models	Demonstrates direct linkage between enzymatic inhibition and disease modification	Doyle et al. (2007)
Comparative homology	42.2% sequence identity with human CTSL; 38% with CTSS; > 70% active‐site residue conservation across human CTSs	Anticipates selectivity constraints during optimisation	Eakin et al. (1992), Brak et al. (2008), Sartori et al. (2019) and Lecaille et al. (2002)

*Note:* Summary of the primary experimental observations that establish cruzain as a tractable and validated drug target, including structural characterisation, genetic/functional essentiality and in vivo pharmacological proof‐of‐concept.

## 3 Cruzain Inhibitors

### 3.1 Why Potency Alone Fails: The K777 Paradigm

K777 is the most advanced CZ inhibitor, and its failure to advance beyond preclinical development illuminates the broader translational pathology. We dissect the K777 trajectory and the sequential scaffold‐rescue strategies it motivated to establish a diagnostic framework for the field.

K777 irreversibly inactivates CZ through Michael addition of the Cys25 thiolate to the vinyl sulfone warhead, forming a β‐thioether sulfone adduct resistant to hydrolytic cleavage (Engel et al. 1998). Enzymatic potency is unequivocal: *K*_*i*_ = 0.2 nM in biochemical assays (Engel et al. 1998). This potency translates into pharmacological proof of concept in vivo: oral administration of K777 produces robust parasitological cure in murine models of acute *T. cruzi* infection, as demonstrated by sustained clearance of parasitaemia relative to untreated controls (Doyle et al. 2007). These findings provide strong pharmacological support for CZ inhibition as a viable therapeutic mechanism.

Despite this compelling efficacy, K777 failed to progress as a clinical candidate. This failure did not arise from inadequate target engagement but from the convergence of multiple scaffold‐intrinsic liabilities. The peptidomimetic backbone imposes severely limited and non‐linear oral exposure, in part due to transporter‐mediated efflux, creating a permeability ceiling that is unlikely to be overcome by formulation strategies alone (Choy et al. 2013). In parallel, the vinyl sulfone warhead functions as a mechanism‐based inhibitor of CYP3A4, introducing a high‐risk drug–drug interaction liability that further constrains systemic exposure (Choy et al. 2013). Repeat‐dose toxicology studies additionally revealed dose‐limiting hepatic and gastrointestinal adverse effects in higher‐order species, effectively narrowing the therapeutic window (Jasinski et al. 2024).

Critically, these liabilities are not independent or sequential hurdles but structurally entangled features of the same molecular architecture. Attempts to mitigate any single limitation generally preserve the others, exemplifying the fundamental constraint of sequential single‐parameter optimisation (SPO).

The medicinal chemistry response to K777 illustrates this pathology in practice. WRR‐483 replaced the P2 phenylalanine with arginine, introducing polarity while preserving enzymatic potency and antiparasitic activity in vitro and in vivo, yet without resolving oral exposure limitations (Engel et al. 1998). Subsequent substitution of arginine with canavanine (WRR‐662), an unnatural amino acid with a lower pK_a_, explicitly targeted the unionised fraction at physiological pH to improve absorption. Further scaffold refinement, including side‐chain homologation and replacement of the terminal phenyl group with a 2‐pyrimidinyl moiety, yielded WRR‐669, which displayed an unusual pH‐dependent dual mechanism: irreversible covalent inhibition at mildly acidic pH and fully reversible non‐covalent inhibition at near‐neutral pH, as confirmed crystallographically (Choy et al. 2013). Each modification addressed a single measurable parameter; none escaped the fundamental exposure constraints imposed by the peptidyl backbone. The trajectory from K777 to WRR‐669 is, in miniature, the trajectory of the entire vinyl sulfone class.

A further dimension of the K777 paradigm is revealed by the enzyme‐to‐organism translation gap. K777 itself retains low‐micromolar activity against intracellular amastigotes, but systematic analyses across broader peptidomimetic and vinyl sulfone series document one‐to‐three orders of magnitude erosion of potency between recombinant CZ assays and whole‐parasite infection assays (Chenna et al. 2020; Pauli et al. 2022). This disconnect reflects the combined effects of parasitophorous vacuole penetration, pH‐dependent intracellular partitioning, and active efflux on the effective drug concentration at the site of parasite replication. Potency measured against the isolated enzyme is therefore a poor predictor of efficacy at the organismal level—a fact already evident in the K777 series and amplified across all subsequent peptidyl vinyl sulfone analogues.

Finally, the selectivity profile of K777 further constrains its therapeutic window. Experimental selectivity data against human CTSs remain strikingly sparse, a gap that is itself a diagnostic marker of the field's potency‐centric bias. The limited available evidence indicates that selectivity achievable with an irreversible warhead targeting a catalytic triad conserved at > 90% across the CZ‐CTS family is intrinsically narrow (Choy et al. 2013; Jasinski et al. 2024). That the most advanced CZ inhibitor reached preclinical toxicology without a systematic CTS selectivity panel highlights the extent of the field's historical potency‐centric emphasis. Achieving meaningful selectivity requires exploiting non‐conserved peripheral subsites, precisely those regions where CZ isoform diversity introduces additional structural uncertainty.

The K777 trajectory thus exemplifies why potency‐first design cannot deliver clinical candidates for intracellular targets.

### 3.2 The Permeability Wall: When Drug‐Likeness Fails to Translate Into Intracellular Exposure

Following the K777 impasse, the field shifted to non‐peptidic, drug‐like scaffolds (hydrazones, thiosemicarbazones, triazines and benzimidazoles) designed to satisfy molecular weight, polar surface area, and chemical stability criteria that peptidyl architectures inherently violate (Duschak and Couto 2009; Ferreira et al. 2014).

However, the non‐peptidic era exposed a second, equally restrictive translational barrier. Across chemically distinct non‐peptidic series, enzymatic potency against CZ frequently failed to translate into sustained antiparasitic activity in cellular or infection‐relevant assays. This enzyme‐to‐cell disconnect has been documented across multiple virtual screening and structure‐based optimisation campaigns (Ferreira et al. 2014; Santos et al. 2023; Wiggers et al. 2013). The consistency of this disconnect points to a systematic exposure problem rather than stochastic assay artefacts.

Effective inhibitors must therefore traverse, sequentially, the host‐cell plasma membrane, the parasitophorous vacuole membrane, and the parasite plasma membrane to accumulate within infected host cells at concentrations sufficient for target engagement. This multi‐layered barrier has been recognised as a dominant determinant of antiparasitic efficacy and is poorly captured by conventional permeability assays or Lipinski‐style heuristics (Rycker et al. 2018; Barrias et al. 2013; Romano et al. 2012).

Non‐peptidic CZ inhibitors frequently illustrate this limitation. Weakly basic or neutral heterocycles optimised for membrane permeability at physiological pH may fail to retain adequate protonation under acidic or compartmentalised intracellular microenvironments, limiting vacuolar retention and effective target engagement. Conversely, compounds engineered to exploit pH‐dependent accumulation may redistribute or undergo efflux upon encountering near‐neutral cytosolic conditions. These physicochemical trade‐offs have been highlighted across multiple non‐peptidic CZ inhibitor series, in which improvements in biochemical affinity did not yield proportional gains in cellular efficacy (Caputto et al. 2012; Ferreira et al. 2014).

Active efflux further compounds this barrier. Several non‐peptidic CZ inhibitor classes have been shown or inferred to be susceptible to host transporter‐mediated clearance, reducing intracellular drug concentrations independently of enzymatic affinity. Such liabilities are often invisible in standard in vitro ADME profiling when assays are conducted outside the context of infected host cells, leading to systematic overestimation of translational potential (García‐Salcedo et al. 2016; Rycker et al. 2018).

Attempts to breach the permeability wall through incremental lipophilicity increases have proven counterproductive. Across non‐peptidic CZ series, cLogP increases of > 1 unit that improved passive permeability simultaneously exacerbated CTS cross‐reactivity and metabolic clearance (Leeson and Springthorpe 2007). This trade‐off is not merely conceptual; it appears as a quantifiable constraint within the CZ‐CTS selectivity landscape (see Section 5.5).

The extent of the disconnect between enzymatic and cellular potency varies substantially across scaffold classes and individual compounds, as illustrated by representative examples compiled in Table S1. The consequences of breaching the permeability barrier become evident when chemistry increases intracellular exposure. Strategies that enhance membrane penetration frequently incur a new penalty (rapid metabolic clearance), revealing the metabolic cliff that dominates the next stage of optimisation. The thiosemicarbazone series discussed in the next section exemplifies this outcome.

### 3.3 The Metabolic Cliff: Thiosemicarbazones as a Cautionary Tale

The permeability wall described in Section 3.2 prompted a further medicinal chemistry pivot. If non‐peptidic scaffolds fail primarily due to inadequate intracellular exposure, then chemotypes that combine sufficient permeability with high intrinsic reactivity towards CZ might restore antiparasitic efficacy. Thiosemicarbazones emerged as an instructive response to this logic. These compounds abandon peptidic recognition elements, retain relatively compact molecular architectures, and incorporate electrophilic functionalities that can engage the catalytic Cys25 residue. From a purely enzymatic perspective, this strategy appears promising.

Indeed, thiosemicarbazones are among the most potent irreversible, non‐peptidic classes reported against CZ. Screening of chemically diverse libraries identified multiple members with submicromolar enzymatic inhibition and measurable selectivity over human CTSs (Du et al. 2002). The mechanistic basis of inhibition is well defined: nucleophilic attack of the Cys25 thiolate on the thiocarbonyl centre generates a covalent thiourea adduct, yielding sustained target engagement under biochemical assay conditions. In isolation, thiosemicarbazones appear to satisfy two constraints that earlier classes failed to meet: a non‐peptidic architecture and high intrinsic potency.

However, this apparent success masks a third and decisive translational barrier. Thiosemicarbazones frequently exhibit unfavourable metabolic stability. The thiocarbonyl electrophilicity that enables Cys25 engagement also renders these compounds susceptible to glutathione conjugation and non‐specific protein adduction, liabilities well documented for this chemotype (Czylkowska et al. 2025; Pelosi 2010; Santoro et al. 2019). Du et al. (2002) noted that trypanocidal activity was rapidly lost in cellular systems despite potent enzymatic inhibition, an observation attributed to metabolic quenching rather than permeability failure. Although quantitative microsomal or hepatic S9 half‐life data have not been reported for the specific CZ‐targeted thiosemicarbazones, the available cellular behaviour is consistent with compounds unlikely to meet the pragmatic > 120 min metabolic‐stability benchmark developed in Section 5. In practical terms, the permeability wall gives way to a metabolic cliff: compounds may reach the parasite, but they do not persist long enough at sufficient intracellular concentrations to sustain antiparasitic activity.

Attempts to mitigate this liability through incremental chemical modification have yielded limited success (Jasinski et al. 2022; Martins et al. 2023). Shielding the electrophilic centre reduces reactivity towards off‐target nucleophiles but simultaneously diminishes on‐target potency. Conversely, enhancing electrophilicity compromises enzymatic inhibition while increasing metabolic stability and safety. These trade‐offs mirror the anticorrelated optimisation landscapes encountered in Sections 3.1 and 3.2. Once again, a sequential single‐parameter strategy appears poorly suited to resolving the trade‐off: rescuing metabolic stability compromises potency, while restoring potency accelerates clearance.

**FIGURE 1:**
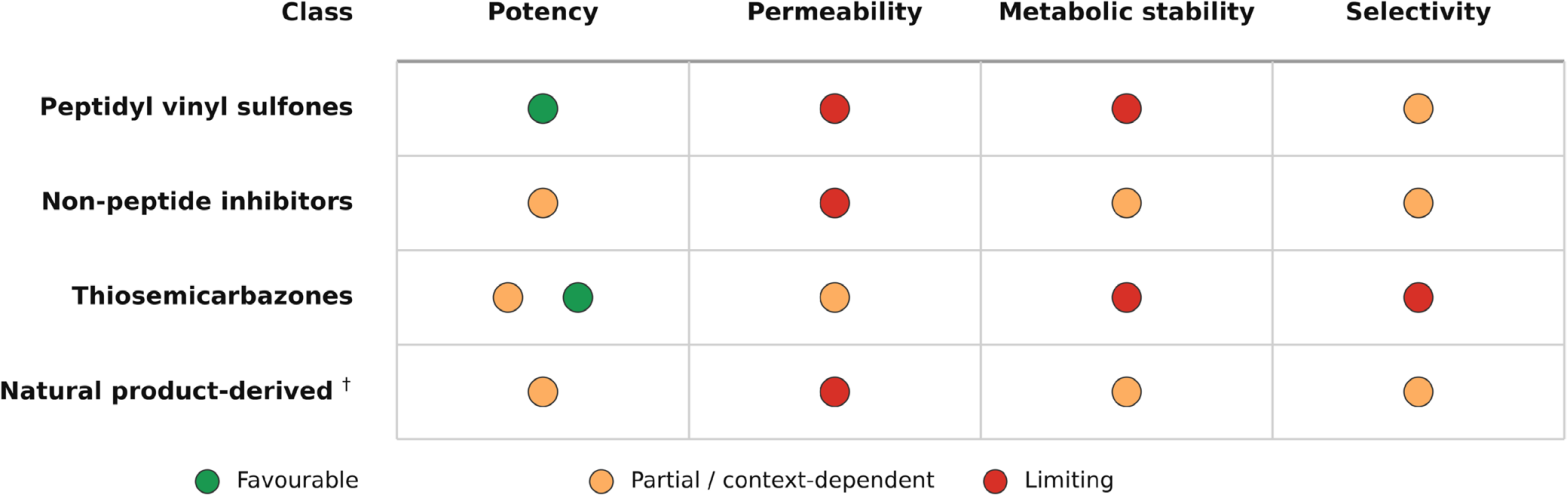
The translational failure landscape of cruzain inhibitor classes. Qualitative heatmap summarising experimentally validated outcomes across major cruzain inhibitor classes. While high enzymatic potency is routinely achieved, each class collapses along at least one translational axis (permeability, metabolic stability or selectivity), preventing clinical progression. Colour coding reflects qualitative assessment based on primary experimental literature; numerical cross‐comparison is not implied. ^†^Natural product‐derived compounds are included for contextual completeness; their limited intrinsic potency and exposure have precluded systematic lead optimisation.

Each inhibitor class thus violates a different translational constraint, summarised qualitatively in Figure 1 and quantitatively in Table 2, together completing a diagnostic triad of failure modes.

**TABLE 2:** Translational barriers in major classes of cruzain inhibitors. Quantitative data from primary sources; qualitative descriptors are used where numerical cross‐comparison across studies is not implied.

Inhibitor class	Enzymatic potency	Cellular activity	CTSL selectivity	MW/cLogP	Dominant translational barrier	Key primary sources
*Peptidyl vinyl sulfones* K777, WRR‐483	*K*_*i*_ = 0.2 nM	Parasitological cure in murine acute models	Not quantified in primary sources; selectivity against human CTS L/B reported as narrow due to conserved active‐site architecture	485/2.8	Oral *F* < 5%; CYP3A4 inhibition; hepatotoxicity in dogs/primates; peptidic backbone	Choy et al. (2013), Doyle et al. (2007) and Jasinski et al. (2024)
*Non‐peptide nitriles* Dipeptidyl nitriles	5–50 nM	Active in cellular assays; in vivo activity demonstrated	Variable	350–450/variable	Residual peptide liability; CYP metabolism	Burtoloso et al. (2017)
*Reversible non‐peptide* Triazines, benzimidazoles	800 nM (triazines); 5–50 μM (benzimidazoles)	Weak or inconsistent; EC_50_ averaging ~6‐ to 20‐fold > IC_50_ (benzimidazoles)	10‐ to 15‐fold (triazines); not tested (benzimidazoles)	320/3.1 (benzimidazoles)	Poor membrane permeability; elevated TPSA; limited intracellular exposure	Ferreira et al. (2014) and Caputto et al. (2012)
*Thiosemicarbazones*	IC_50_ = 380 nM	Trypanocidal at μM concentrations; rapid loss of activity in cellular systems	~10‐fold vs. CTSL	285/2.9	Metabolic instability (rapid loss of cellular activity consistent with short microsomal *t*_1/2_; quantitative data not reported); nonspecific covalent reactivity; low selectivity	Nunes et al. (2025) and Siqueira‐Neto et al. (2018)
*Natural‐product‐derived* Flavonoids, alkaloids, triterpenes	15–100 μM	Weak or absent in vivo translation	Unknown/not tested	Variable	Insufficient potency for lead optimisation; structural complexity; PAINS liability	Siles et al. (2006) and Ndjonka et al. (2013)

*Note:* Evidence quality varies across rows: *K*_*i*_ values for K777 and IC_50_ for thiosemicarbazones derive from primary experimental assays, while selectivity estimates for peptidyl vinyl sulfones and natural‐product classes are inferred from limited or series‐level data. Direct numerical comparison across classes should therefore be interpreted with caution.

Together, Figure 1 and Table 2 map this diagnostic triptych. The consistent failure of all three classes under SPO logic establishes the empirical basis for the MPO framework formalised in Section 5.

## 4 Computational Methods: Precision Without Translation

The field's computational investment has been substantial: molecular docking, molecular dynamics (MD), free‐energy perturbation (FEP), and machine‐learning‐based QSAR (ML‐QSAR) have all been applied to CZ with increasing technical sophistication. Yet this growing computational sophistication has not been accompanied by clinical translation. The limitation may lie less in computational precision than in objective specification: all four methodologies have been predominantly deployed to optimise binding free energy (Δ*G*_bind_ or its surrogates) while the multiparametric determinants of intracellular exposure remain unmodelled.

**FIGURE 2:**
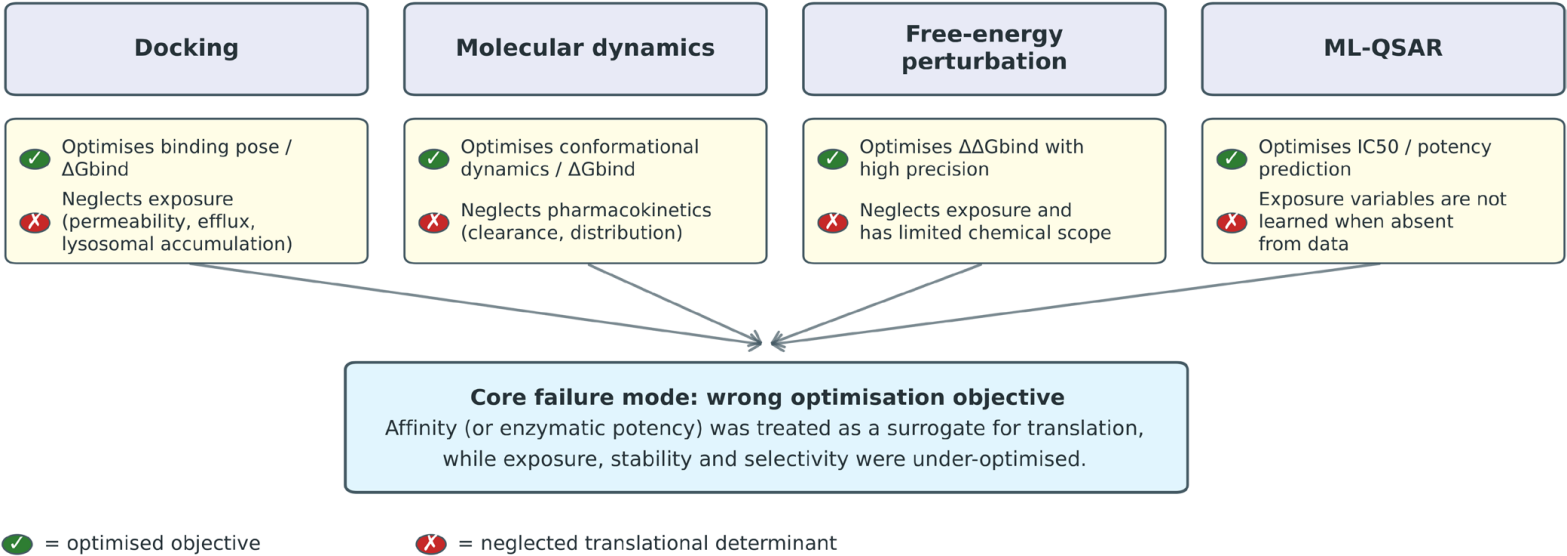
Computational precision without translation in cruzain inhibitor discovery. Molecular docking, molecular dynamics, free‐energy perturbation and machine‐learning‐based QSAR have achieved increasing precision in modelling ligand‐cruzain interactions and predicting binding affinity. However, all methods converge on the same structural limitation: optimisation objectives centred on binding energy or enzymatic potency, while intracellular exposure, metabolic stability, and selectivity remain unmodelled or underrepresented. This misspecified optimisation target explains why computational sophistication has not translated into clinical candidates. It motivates the need for an explicitly multiparametric optimisation (MPO) framework aligned with intracellular exposure and translational constraints.

Figure 2 illustrates this convergent failure mode. Despite their methodological diversity, all four approaches share two critical blind spots: (i) none models membrane traversal, intracellular trafficking, lysosomal accumulation, or metabolic turnover; and (ii) all rely on implicit physicochemical assumptions (most critically, the choice of enzymatic protonation state) that become systematically invalid under infection‐relevant conditions.

### 4.1 The Shared Failure Mode: Binding Affinity as a Misspecified Surrogate for Translation

For intracellular parasitic targets, binding affinity is a necessary but subordinate condition for efficacy. The dominant determinants of therapeutic outcome are whether a compound can reach the parasite, persist in the relevant intracellular compartment, and maintain sufficient free concentration over time without incurring prohibitive toxicity. Computational methods that optimise Δ*G*_bind_ while ignoring these variables will, by construction, prioritise compounds that fail, irrespective of the precision they achieve within their defined objective space.

This is the broad pattern observed across the CZ computational literature. Each major methodology has succeeded on its own terms, but with limited detectable impact on translational progress:

1. Structure‐based docking, the earliest computational approach applied to CZ, successfully rationalised ligand binding modes and identified key interactions within the S2 subsite (McGrath et al. 1995; Ferreira et al. 2014). Docking predictions correlated well with enzymatic potency, reinforcing confidence in the approach. However, docking intrinsically evaluates affinity under idealised conditions: a rigid or semi‐flexible protein, an implicit solvent environment, and no representation of membrane traversal, intracellular trafficking, or pH‐dependent partitioning. Docking scores optimise Δ*G*_bind_ at the active site while remaining blind to whether a compound can reach that site in sufficient concentration.
2. Molecular dynamics (MD) simulations extended docking by incorporating protein flexibility, solvent dynamics, and time‐resolved ligand‐protein interactions. Applied to CZ, MD clarified the dynamic behaviour of the S2 and S3 subsites, the stabilisation of covalent adducts, and the enthalpy‐driven nature of ligand binding (Choy et al. 2013). Yet MD remains fundamentally local in scope: simulations are conducted on nanosecond‐to‐microsecond timescales in homogeneous aqueous environments and do not model membrane permeation, lysosomal accumulation, active efflux, or metabolic turnover.
3. Free‐energy perturbation (FEP) represents the most quantitatively rigorous approach for predicting relative binding free energies within congeneric series, demonstrating strong agreement with experimental affinity data in protease systems (Wang et al. 2015). However, FEP operates within a narrowly defined chemical space and optimises a single objective: ΔΔ*G*_bind_. In the CZ context, incremental affinity gains achieved through FEP‐guided optimisation are frequently eclipsed by losses in permeability, selectivity or metabolic stability (yielding high‐confidence predictions that are orthogonal to the parameters governing clinical viability).
4. Machine‐learning‐based QSAR (ML‐QSAR) has been applied to enzymatic inhibition datasets with reasonable internal validation performance (Gonçalves et al. 2023). However, the underlying datasets are typically small (< 350 compounds in the largest CZ‐specific training set), chemically heterogeneous, and dominated by biochemical rather than cellular endpoints. Models trained primarily on enzymatic IC_50_ values inevitably learn to predict potency rather than translation. Exposure‐related properties (intracellular accumulation, efflux susceptibility, metabolic clearance) are either absent from training data or represented indirectly through crude physicochemical proxies.

The convergence is striking: four methodologically distinct approaches, spanning physics‐based and data‐driven paradigms, converge on the same translational outcome because they share the same misspecified objective function. This diagnosis does not indict the methods themselves but rather the strategic framework within which they have been deployed.

### 4.2 pH‐Protonation Bias: A Systematic Error Across Computational Workflows

**FIGURE 3:**
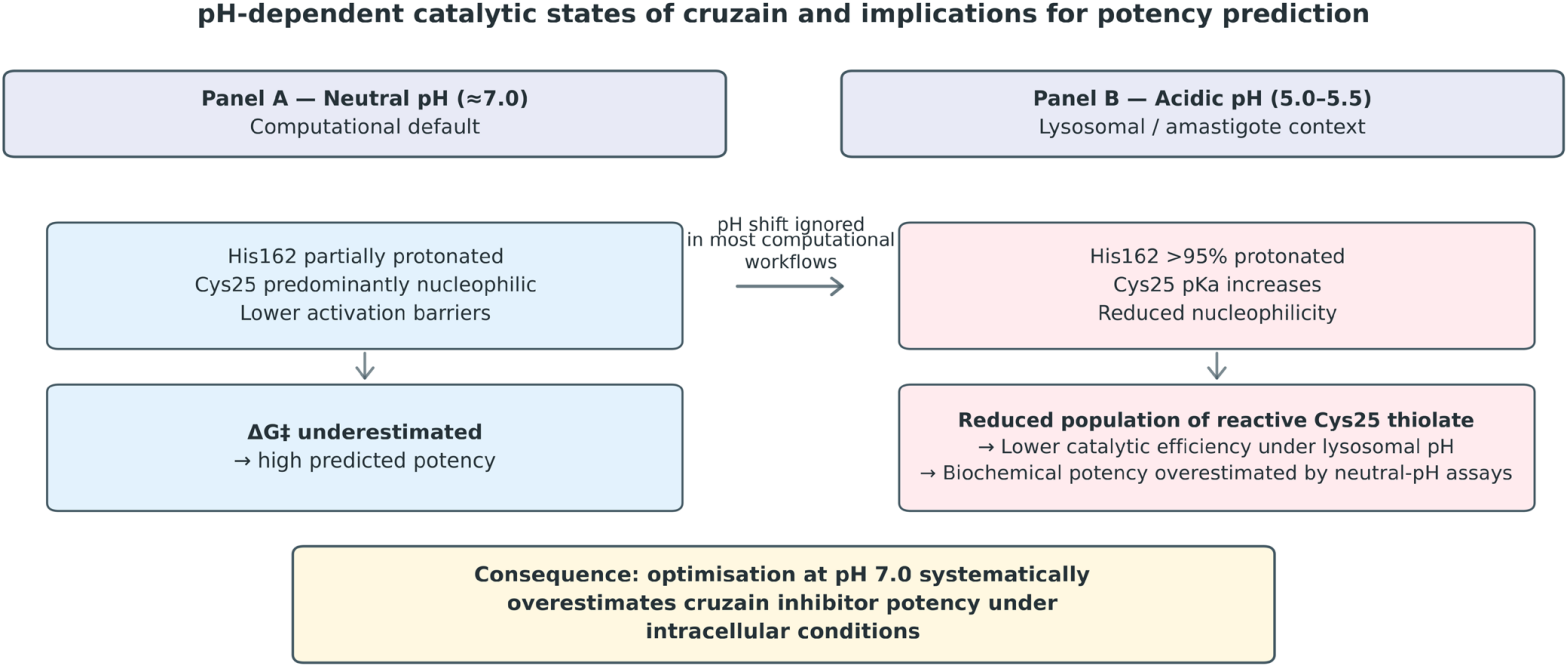
pH‐dependent catalytic states of cruzain and implications for computational potency prediction. Conceptual representation of cruzain catalytic states under neutral pH conditions typically employed in computational workflows (Panel A) versus acidic lysosomal conditions relevant to intracellular amastigotes (Panel B). At pH ~7.0, standard workflows commonly assign protonation states that favour a catalytic geometry compatible with enhanced Cys25 nucleophilicity. Result in lower predicted activation barriers and overestimated inhibitor potency. Under acidic conditions (pH 5.0–5.5), His162 is predominantly protonated, and the effective pK_a_ environment of Cys25 is altered, changing the electronic context for inhibitor binding and covalent reactivity. These protonation‐state differences can bias potency prediction and, for pH‐sensitive inhibitors such as WRR‐669, may assign an inhibitory mechanism that does not correspond to the infection‐relevant compartment.

Beyond the shared misspecification of the objective function, computational workflows applied to CZ embed a second, often‐overlooked source of systematic error: the assumption of neutral‐pH protonation states for the catalytic triad. As summarised in Figure 3, modelling CZ at pH ~7.0 (a standard default in docking, MD and FEP workflows) fails to capture the acidic microenvironment experienced by intracellular amastigotes, where the enzyme operates at pH 5.0–5.5.

The consequences are mechanistically specific. At neutral pH, His162 is only partially protonated and Cys25 retains substantial nucleophilic character, yielding the catalytic geometry implicitly assumed by standard computational workflows. Under lysosomal conditions (pH 5.0–5.5), His162 is predominantly protonated and the effective pKa of Cys25 is expected to rise, reducing the population of the reactive thiolate form (Figure S4). These pH‐dependent shifts in catalytic triad protonation alter the electronic environment governing inhibitor binding and covalent reactivity, thereby weakening the assumption that affinity measured or modelled under standard neutral‐pH conditions is transferable to infection‐relevant acidic compartments. The clearest experimental illustration is the WRR‐669 series, which undergoes a mechanistic switch from irreversible covalent inhibition at pH 5.5 to fully reversible non‐covalent binding at pH 8.0, as confirmed crystallographically (Choy et al. 2013; Jasinski et al. 2024). Thus, a computational workflow that models CZ‐inhibitor binding only at neutral pH does not merely introduce numerical uncertainty; for pH‐sensitive inhibitors such as WRR‐669, it can assign the wrong inhibitory mechanism.

No QM/MM study has yet quantified the activation free‐energy shift between neutral and lysosomal pH regimes for the natural catalytic cycle of CZ (Figure S4), and the magnitude of this bias therefore remains unknown. Nevertheless, its translational implication is clear: protonation state must be treated as an explicit model variable rather than a default preprocessing choice. Otherwise, even technically rigorous docking, MD, or FEP workflows can produce high‐confidence potency predictions under conditions that do not represent the intracellular compartment in which CZ inhibition must occur. This protonation‐state bias likely acts additively with the exposure and permeability barriers diagnosed in Section 3.

### 4.3 Repositioning Computational Tools: From Optimisers to Multiparametric Filters

The diagnosis above does not imply that computational methods are irrelevant to CZ drug discovery. It implies that their role must be fundamentally reframed: from single‐objective optimisers to multiparametric filters and integrators.

Within an MPO framework (formalised in Section 5), each methodology retains value when deployed for the right purpose. Docking and MD simulations are best repurposed as exclusionary filters: they can efficiently eliminate compounds that cannot plausibly engage CZ or that rely on interactions incompatible with selectivity constraints, without claiming to rank‐order translational potential. FEP is most productively deployed within tightly defined congeneric series to prioritise substitutions that improve affinity without degrading permeability or stability metrics that already meet threshold criteria (Wang et al. 2015). ML‐QSAR models become most useful when trained on multiparametric endpoints, integrating biochemical activity with cellular efficacy, metabolic stability, and transporter liability, rather than on enzymatic potency alone. Such models can identify regions of chemical space that simultaneously satisfy multiple constraints and must be used to reduce chemical space rather than to predict absolute performance (Gonçalves et al. 2023).

Critically, computational workflows must also be corrected for the pH‐protonation bias identified in Section 4.2. Prospective studies should model CZ under acidic conditions (pH 5.0–5.5) rather than the neutral‐pH defaults that have dominated the field. Without this correction, even multiparametrically aware pipelines will be systematically biased at the potency‐prediction stage.

In this reframed role, computation no longer promises to deliver the ‘best’ compound. Instead, it enables efficient navigation of constrained optimisation landscapes by identifying candidates that are good enough across all relevant dimensions, the feasibility envelope concept developed in Section 5.5.

## 5 A Multiparametric Optimisation (MPO) Framework for Cruzain Inhibitors

**FIGURE 4:**
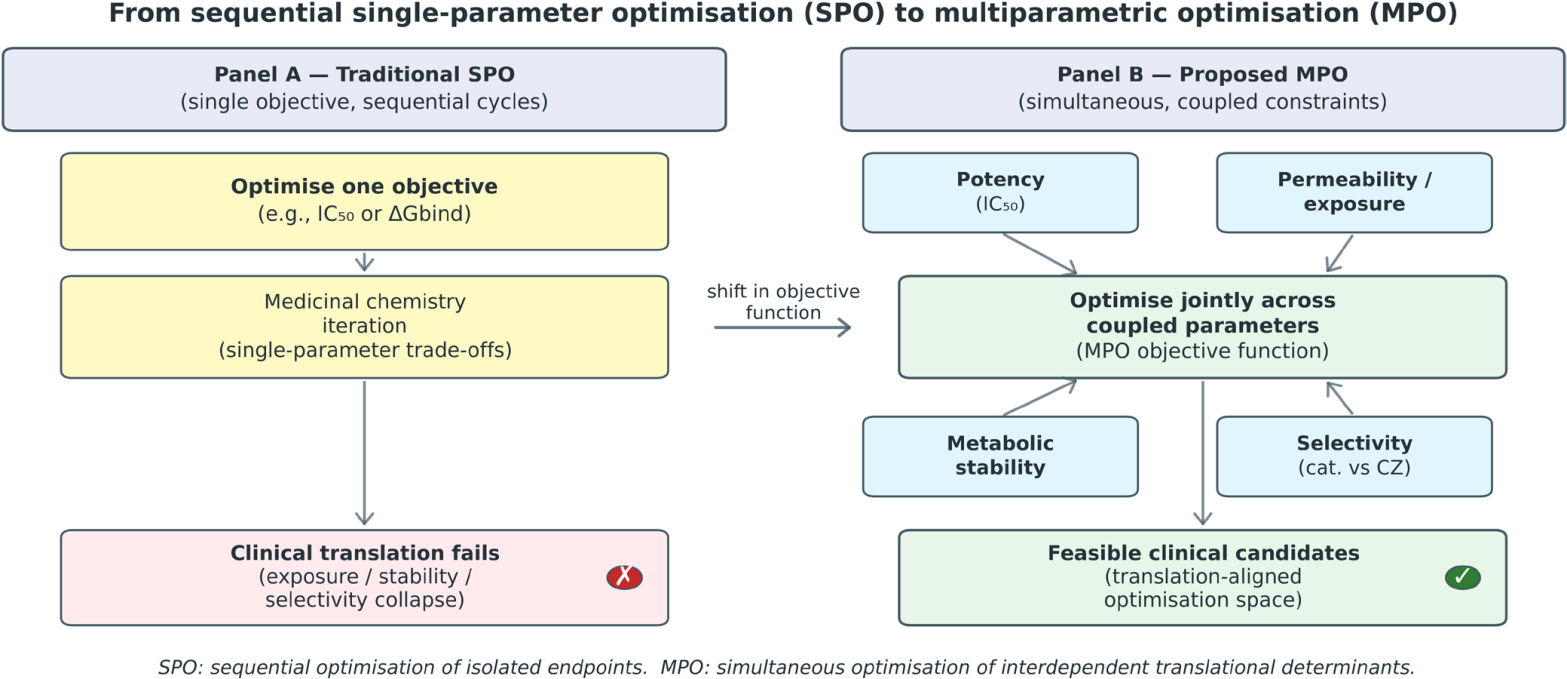
Transition from sequential single‐parameter optimisation (SPO) to a multiparametric optimisation (MPO) framework for cruzain inhibitor discovery. Conventional optimisation strategies in the cruzain field have prioritised single objectives (most commonly enzymatic potency or binding affinity) addressed sequentially through medicinal chemistry cycles. As demonstrated throughout Sections 3 and 4, this approach systematically neglects the coupled constraints governing intracellular exposure, metabolic stability, and selectivity, leading to translational failure despite high biochemical potency. The proposed MPO framework replaces this sequential logic with simultaneous optimisation across potency (IC_50_), permeability, metabolic stability, and cathepsin selectivity. By treating these parameters as interdependent rather than independent variables, MPO defines a feasible optimisation space in which computational and experimental methods can be aligned with clinical translation rather than solely on binding affinity.

The diagnostic triad established in Sections 3 and 4 demands a fundamentally different optimisation logic. Figure 4 formalises the transition from sequential single‐parameter optimisation (SPO) to a multiparametric optimisation (MPO) framework, in which potency, permeability, metabolic stability, and CTS selectivity are treated as coupled rather than sequential design constraints.

Table 3 operationalises this framework, specifying the minimal parameter set, recommended assays, and proposed decision criteria required to align design and screening with determinants of intracellular exposure.

**TABLE 3:** Proposed multiparametric optimisation (MPO) criteria for translation‐ready cruzain inhibitors.

MPO dimension	Why it matters	Suggested assay/metric	Proposed decision criterion^a^	Derivation basis	Notes/caveats
Enzymatic potency	Ensures target engagement at feasible exposure levels	CZ IC_50_ (biochemical assay)	IC_50_ < 100 nM	Back‐calculated from K777 paradigm: K777 (*K*_*i*_ = 0.2 nM) achieved in vivo cure but failed on exposure; ~100 nM represents the minimum potency at which realistic intracellular concentrations (accounting for ~10–100‐fold enzyme‐to‐cell attenuation documented across scaffold classes; Section 3) can sustain target engagement	Necessary but insufficient alone
Selectivity	Minimises off‐target CTS inhibition and toxicity	Panel vs. CTSL, CTSB, CTSS	> 100‐fold selectivity	Derived from structural constraint: CZ‐CTS S2 subsite conservation (> 60% residue identity; Section 2) establishes a thermodynamic floor on achievable selectivity; 100‐fold represents a pragmatic minimum consistent with acceptable therapeutic windows in cysteine protease inhibitor programmes (Turk et al. 2012)	Active‐site conservation limits margin
Lipophilicity/exposure	Governs membrane permeability and intracellular accumulation	LogD_7.4_ (shake‐flask or potentiometric)	LogD_7.4_ > 1.5	General anti‐infective literature: LogD_7.4_ > 1.5 reflects the minimum lipophilicity empirically associated with adequate membrane permeation for intracellular anti‐infective agents requiring multi‐barrier traversal (Leeson and Springthorpe 2007; Rycker et al. 2018)	Excessive lipophilicity penalises solubility
Metabolic stability	Determines the duration of intracellular exposure	Microsomal *t*_1/2_	> 120 min^b^	Pragmatic benchmark from general medicinal chemistry practice: microsomal *t*_1/2_ < 30–60 min is typically associated with rapid clearance and poor intracellular exposure for anti‐infective agents; > 120 min reflects the threshold above which sustained intracellular concentrations become achievable over standard dosing intervals (Pauli et al. 2022). The thiosemicarbazone failure (Section 3.3) provides a negative exemplar	Species differences must be considered
Efflux liability	Limits the effective intracellular concentration	P‐gp/MDR1 assays	Low to moderate risk	Inferred from CZ biology: multi‐barrier architecture (Section 5.5) amplifies the impact of efflux on effective intracellular concentration; threshold is qualitative pending CZ‐specific efflux data	Context‐dependent relevance
pH‐dependent partitioning	Relevant to parasite compartmentalisation	pK_a_/distribution modelling	Compatible with pH 5–7.4	Derived from CZ biology: CZ operates across pH 5.0–7.4 (Section 2); compounds must retain adequate unionised fraction across this range to maintain binding competence and avoid pH‐trapping outside the target compartment	Proxy measures only

*Note:* Operational framework defining the minimal set of coupled parameters that must be optimised simultaneously to enable clinical translation.

^a^ These thresholds represent proposed benchmarks for this framework, not experimentally validated decision criteria, and are intended as a starting point for prospective validation.

^b^ Threshold values represent proposed benchmarks informed by general medicinal chemistry practice and the translational requirements of intracellular targets. They are not experimentally validated decision criteria for cruzain inhibitors and may require adjustment depending on compound class, dosing strategy, and pharmacokinetic context.

The derivation basis for each threshold is specified in Table 3. Three categories of evidence inform these criteria: (1) back‐calculation from the CZ failure landscape; thresholds that could have predicted the known failure of K777, thiosemicarbazones, or non‐peptide scaffolds are anchored in the retrospective diagnostic presented in Section 3; (2) general anti‐infective medicinal chemistry literature; benchmarks established for intracellular targets with comparable barrier architectures; and (3) CZ‐specific structural constraints; parameters dictated by the biology of the target (pH range, CTS homology, multi‐barrier localisation). None of these thresholds has been prospectively validated for CZ inhibitors. Their value lies not in precision but in the explicit recognition that translation requires simultaneous satisfaction of coupled constraints, a principle that the historical record of CZ inhibitor discovery validates by repeated negative example.

The MPO criteria formalised in Sections 5.1, 5.3 raise two prior questions, addressed in turn: whether the anticorrelation constraints documented here are CZ‐specific or generic to antiparasitic drug design (Section 5.4), and whether the apparent anticorrelation between potency and exposure is navigable in principle (Section 5.5). The answers bear directly on the urgency and scope of the proposed framework.

### 5.1 From Sequential Optimisation to Constraint Satisfaction

For CZ specifically, improving any single parameter predictably tightens constraints on others (Leeson and Springthorpe 2007). This anticorrelated landscape, diagnosed empirically in Sections 3.1, 3.3 and analysed mechanistically in Section 5.5, demands a fundamentally different optimisation logic.

An MPO framework reframes optimisation as a constraint satisfaction problem. Rather than maximising a single objective, candidate compounds must simultaneously satisfy a defined set of minimum criteria across orthogonal dimensions. Compounds that fail any single constraint are deprioritised, regardless of excellence along other axes. This shift transforms medicinal chemistry decision‐making from incremental optimisation to strategic triage.

### 5.2 Defining Viability Thresholds for Cruzain Inhibitors

The criteria proposed below should be understood as heuristic starting points rather than experimentally validated decision rules. Each threshold is anchored either in (i) retrospective back‐calculation from the CZ failure landscape (Section 3), (ii) general anti‐infective medicinal chemistry practice for intracellular targets, or (iii) CZ‐specific structural constraints (pH operating range, CTS homology, multi‐barrier localisation). None has been prospectively validated for CZ inhibitors, and all will require refinement as multiparametric datasets expand. Their value lies not in numerical precision but in shifting the optimisation objective from potency maximisation to simultaneous constraint satisfaction.

Based on the cumulative evidence reviewed herein, we identify four parameters as central for CZ inhibitor viability: enzymatic potency, intracellular exposure, metabolic stability, and selectivity over host CTS. While precise thresholds will evolve as datasets expand, the current literature supports provisional criteria that are both conservative and experimentally grounded.

The proposed metabolic stability threshold (*t*_1/2_ > 120 min) is intended as a pragmatic benchmark rather than a strict decision rule. This value reflects general medicinal chemistry practice for intracellular anti‐infective agents, where microsomal or cellular half‐lives below 30–60 min are typically associated with rapid clearance and poor intracellular exposure, while half‐lives exceeding approximately 2 h are more likely to sustain therapeutically relevant concentrations over dosing intervals. Importantly, the threshold is not presented as an experimentally validated cutoff for CZ inhibitors, but as a provisional guideline to anchor multiparametric optimisation and enable systematic triage across potency, exposure and stability.

Enzymatic potency remains necessary, but must be bound. We suggest that a sub‐100 nM biochemical IC_50_ range provides a pragmatic starting point for potency triage to ensure that reasonable intracellular concentrations translate into target engagement (Engel et al. 1998). However, potency beyond this range confers diminishing returns if exposure is inadequate.

Selectivity over human CTSs L, B, and S is essential to avoid mechanism‐based toxicity. Given that the catalytic triad and oxyanion hole are structurally superimposable across clan CA cysteine proteases, and that S2 subsite residues show substantial conservation with human CTSL and CTSB (Gillmor et al. 1997; Lecaille et al. 2002), a selectivity margin of at least two orders of magnitude appears to be a reasonable minimum, pending compound‐specific pharmacokinetic context for advancing irreversible or slowly reversible inhibitors.

Intracellular exposure is the dominant translational variable. Compounds must demonstrate the ability to accumulate and persist within infected host cells at concentrations sufficient to inhibit CZ in situ. While no single in vitro assay captures this property, convergence of evidence from cellular EC_50_ values, infection‐relevant assays, and transporter liability assessments provides a pragmatic proxy (Rycker et al. 2018).

Metabolic stability defines the temporal dimension of exposure. Rapid clearance undermines even optimally permeable compounds, as illustrated by thiosemicarbazones. As outlined above, microsomal or cellular *t*_1/2_ exceeding ~2 h provides a pragmatic exposure‐duration benchmark.

These criteria are not independent targets but coupled constraints. A compound that excels along three dimensions but fails the fourth is unlikely to translate.

Computational methods repurposed as exclusionary filters and multiparametric integrators (Section 4.3) become the natural screening layer against these coupled constraints. Docking and MD eliminate compounds incompatible with target engagement; FEP prioritises congeneric substitutions that preserve exposure and stability thresholds already met; and ML‐QSAR models trained on multiparametric endpoints (rather than enzymatic potency alone) reduce chemical space towards the feasibility envelope formalised in Section 5.5. In this reframed role, computation no longer delivers “the best” compound; it identifies candidates that are good enough along all critical axes simultaneously.

### 5.3 Strategic Implications for Future Cruzain Programmes

Adopting an MPO framework imposes a disciplined shift in project strategy. Large‐scale synthesis campaigns aimed at maximising potency become counterproductive. Instead, early integration of exposure and stability screens is mandatory, even at the cost of discarding highly potent molecules. Progress is measured not by record‐breaking IC_50_ values but by the emergence of compounds that remain viable across all constraints.

### 5.4 Cruzain‐Specific Challenges in the Context of Antiparasitic Drug Design

The translational failures documented for CZ inhibitors raise a question of broader significance: are the anticorrelated optimisation landscapes described in this review a peculiarity of CZ, or do they represent a generic liability of drug design against intracellular parasitic targets? The answer is nuanced—the problem is partially generic but critically exacerbated by features specific to CZ and its biological context.

The generic component is well recognised. Any compound designed to inhibit an enzyme sequestered within a parasite that itself resides within a host cell must traverse multiple biological membranes, resist hepatic first‐pass metabolism, and maintain sufficient free‐drug concentration at the site of action. The clinical failure of CYP51 inhibitors for CD (posaconazole and fosravuconazole both showed initial parasite clearance followed by frequent parasitological relapse, whilst benznidazole sustained negative PCR results) (Molina et al. 2014; Torrico et al. 2021) confirms that the translational barrier in CD is not exclusive to CZ. However, several lines of evidence suggest that the CYP51 failures were driven primarily by incomplete parasite eradication, possibly involving quiescent or slowly replicating amastigote subpopulations resistant to cytostatic CYP51 inhibition (Molina et al. 2014; Torrico et al. 2021), rather than by physicochemical liabilities of the inhibitors, which had already achieved adequate systemic exposure through prior development as antifungal agents.

Three structural and biological features make the anticorrelation problem particularly severe for CZ. First, subcellular localisation: CZ resides predominantly in the lysosome of the intracellular amastigote. An orally administered inhibitor must therefore cross six successive biological barriers (the intestinal epithelium, the hepatic first‐pass barrier, the host cell plasma membrane, the parasitophorous vacuole membrane, the parasite plasma membrane, and the lysosomal membrane) with the four intracellular segments imposing cumulative selection for lipophilicity. This is substantially more demanding than the barrier architecture faced by falcipain inhibitors, where the target resides in the food vacuole of *Plasmodium* within the enucleated erythrocyte, which lacks active endocytic machinery and is additionally modified by parasite‐derived permeation pathways (Sajid and McKerrow 2002; Rycker et al. 2018). Second, structural homology with host CTSs: CZ shares only partial overall sequence identity with human CTSs, but the active‐site region is markedly more conserved. Consequently, meaningful selectivity must be extracted from subtle differences in the S2 and S3 subsites rather than from broad topological divergence. In practice, however, the substituents that improve selectivity frequently increase polarity and molecular size, thereby reducing permeability and directly opposing the physicochemical profile required for intracellular target access (Cianni et al. 2019; Sartori et al. 2019; Gillmor et al. 1997). This creates a selectivity‐permeability trade‐off cycle: every polar or charged substituent added to exploit non‐conserved S2/S3 residues for selectivity simultaneously increases molecular weight and topological polar surface area (TPSA), driving the compound away from the lipophilicity window required to traverse four sequential lipid barriers. Empirically, this is reflected in the observation that the most selective CZ inhibitors in the literature are also the least permeable (see Section 3.2 and Table S1). Third, target redundancy: *T. cruzi* expresses at least four cruzipain (CZP) subtypes organised into two genomic families (Santos et al. 2025). Family I (including CZ/CZP1, the standard drug design target) predominates in epimastigotes, whereas Family II members (CZP2, CZP3, CZP4), which differ in key active‐site residues, are preferentially expressed in trypomastigotes and amastigotes, the stages most relevant to disease (Santos et al. 2025). Inhibitors optimised exclusively against recombinant CZ may therefore show attenuated efficacy against the isoform repertoire encountered in situ, requiring higher effective intracellular concentrations than predicted from single‐isoform biochemical IC_50_ values.

Instructive contrast is provided by fexinidazole, a nitroimidazole that reached Phase 2 for CD. Its target‐agnostic, redox‐cycling mechanism bypasses the potency‐selectivity trade‐off entirely but substitutes a different anticorrelation: dose‐toxicity coupling driven by the same non‐selective reactivity that enables broad‐spectrum antiparasitic activity. An initial dose‐finding trial (NCT02498782) was interrupted after transient grade 3–4 neutropenia at higher doses, despite rapid parasitological clearance (Torrico et al. 2023). A subsequent low‐dose trial (FEXI‐12) achieved acceptable safety but only transient parasitological effect; parasite load rebounded within 10 weeks of treatment cessation (Pinazo et al. 2024). The fexinidazole trajectory thus illustrates that anticorrelation is a structural feature of antiparasitic drug design, not a peculiarity of target‐based approaches, but the nature of the anticorrelated parameters differs. For CZ, it is potency‐selectivity‐permeability; for fexinidazole, it is dose‐efficacy‐toxicity. The MPO framework proposed here addresses the former; the latter requires a fundamentally different (pharmacokinetic) solution.

### 5.5 Navigating Anticorrelation: The Feasibility Envelope for Cruzain Inhibitor Optimisation

A critical question arises from the analysis presented in this review: if potency and drug‐likeness are anticorrelated in CZ inhibitor space, can multiparametric optimisation genuinely resolve this tension, or does it merely reframe an intractable problem? We argue that the anticorrelation documented across three decades of CZ inhibitor development appears to be predominantly empirical, reflecting the chemical space actually sampled under a single‐parameter paradigm, rather than an absolute thermodynamic constraint, though a partial thermodynamic component cannot be formally excluded. A thermodynamic anticorrelation would imply that the molecular features required for high‐affinity binding to the CZ active site are physically incompatible with oral bioavailability, membrane permeation, and metabolic stability. An empirical anticorrelation, by contrast, reflects the historical trajectory of a field that systematically explored chemical space under a single‐parameter paradigm.

Three lines of evidence support the empirical interpretation. First, the chemical space explored for CZ inhibition remains comparatively narrow relative to other protease targets of similar age: of the 215 unique inhibitors curated from 668 IC_50_ records (CHEMBL3563; Methods S1), the majority cluster within peptidyl scaffolds or a small number of non‐peptide chemotypes, whilst fragment‐like space (MW ≤ 300 Da, cLogP ≤ 3) remains largely unexplored. Second, ligand efficiency analysis of the curated CHEMBL3563 dataset (*n* = 215; see Methods S1 and Figure S5) shows that potency tracks molecular weight substantially more strongly than ligand efficiency (Pearson r(MW, pIC_50_) = +0.610 versus r(LE, pIC_50_) = −0.213; both *p* ≤ 1.6 × 10^−3^). Stratification by potency tertile reveals that the highest‐potency subset is reached, on average, by adding ~18 heavy atoms relative to the mid‐potency subset while ligand efficiency simultaneously falls from 0.44 to 0.31 kcal/mol per heavy atom, a pattern indicating that incremental potency has been purchased through molecular growth rather than through more efficient engagement of the active site (Hopkins et al. 2014; Leeson and Springthorpe 2007). Third, successful precedents from adjacent fields demonstrate that apparent anticorrelation can be overcome when MPO is applied ab initio. The evolution of HIV‐1 protease inhibitors from first‐generation peptidomimetics with severe metabolic liabilities to darunavir (*K*_*i*_ = 16 pM, oral bioavailability > 80% with ritonavir boosting) illustrates that protease inhibitor space is navigable when design is multiparametric from inception (Ghosh et al. 2012; Rittweger and Arastéh 2007). Similarly, the CNS MPO desirability tool demonstrated that deliberate navigation of property space increased clinical candidate survival by shifting design towards less lipophilic chemical matter whilst maintaining adequate target engagement (Wager et al. 2016).

The resolution of the apparent paradox lies in recognising that MPO does not promise to eliminate anticorrelation; rather, it provides a framework to navigate within it. The key conceptual shift is from seeking compounds that are optimal in every dimension to identifying compounds that satisfy simultaneous minimum thresholds across all critical parameters—what we term the feasibility envelope. For CZ inhibitors, a realistic feasibility envelope might be defined by the concurrent criteria outlined in Table 3. Critically, none of these individual thresholds demands extreme optimisation; the challenge lies exclusively in their simultaneous satisfaction.

The feasibility envelope concept acknowledges that anticorrelation is partially intrinsic to the CZ system, driven by the four sequential lipid barriers separating the host cytoplasm from the lysosomal target (host plasma membrane, parasitophorous vacuole membrane, parasite plasma membrane, and parasite lysosomal membrane; see Section 5.4 for the complete absorption‐to‐target barrier architecture, including the upstream intestinal and hepatic barriers). However, ‘partially intrinsic’ is not ‘absolute’. The existence of compounds such as the thiosulfonate TSO‐3 (IC_50_^CZP^ = 2.7 μM, 96% infection reduction at 10 μM, no cytotoxicity at 50 μM; Cerutti et al. 2025) and the quinazoline 1s (*K*_*i*_ = 1.9 μM, EC_50_ = 0.370 μM against *T. cruzi* , SI > 27; Silva et al. 2021)—compounds achieving moderate potency with acceptable selectivity and cellular translation—suggests that the feasibility envelope is not empty. The framework thus reframes the translational failure of CZ inhibitors not as evidence of target invalidity, but as a plausible consequence of a design philosophy that treated potency as the dominant objective function. Whether the envelope contains clinical candidates remains an empirical question—one that can only be answered by systematically exploring it.

## 6 Outstanding Questions and Translational Agenda

The diagnostic framework established above identifies the specific barriers to CZ inhibitor translation. What remains unresolved are the empirical hierarchies among competing parameters and the experimental strategies required to validate them. The following open questions define a translational agenda that can convert diagnostic insight into actionable progress.

- Does pH‐dependent intracellular partitioning dominate cellular efficacy over passive permeability?
  Most CZ inhibitor discovery efforts have relied on neutral‐pH permeability assays as proxies for intracellular exposure. However, CZ operates across a compartmentalised intracellular landscape in which pH varies substantially between parasite stages and subcellular locations. This raises a fundamental translational question: whether pH‐dependent ionisation and intracellular partitioning better predict cellular efficacy than passive permeability metrics alone.
  ○ Translational implication: If pH‐dependent partitioning is a dominant determinant of intracellular exposure, permeability optimisation strategies that ignore ionisation dynamics may systematically misprioritise compounds.
  ○ Experimentally testable approach: Matched analogue series in which pK_a_ is modulated independently of lipophilicity could be evaluated for intracellular accumulation and cellular EC_50_ under infection‐relevant conditions.
- Can early multiparametric triage reduce attrition without sacrificing efficacy?
  Conventional hit‐to‐lead workflows in CZ programmes prioritised enzymatic potency and deferred exposure and stability constraints to later stages of optimisation. The MPO framework predicts that this sequencing is intrinsically inefficient for intracellular targets and that early multiparametric filtering should compress chemical space substantially while preserving translational potential.
  ○ Translational implication: Early application of MPO criteria may reduce downstream attrition and resource expenditure without compromising the probability of in vivo efficacy.
  ○ Experimentally testable approach: Prospective comparison of hit triage pipelines with and without early MPO filtering, benchmarking attrition rates, chemical diversity retention, and progression to in vivo models.
- Is K777 toxicity driven by off‐target cathepsin inhibition or scaffold‐intrinsic liabilities?
  K777 remains the most advanced CZ inhibitor to date, yet its development was terminated by toxicity in higher‐order species. Whether this outcome reflects off‐target CTS inhibition or intrinsic liabilities of the peptidyl vinyl sulfone scaffold remains unresolved. This distinction is decisive for assessing whether covalent warheads of this class remain viable.
  ○ Translational implication: If toxicity is driven predominantly by scaffold‐intrinsic metabolic or electrophilic reactivity, selectivity optimisation alone will be insufficient to rescue the chemical series.
  ○ Experimentally testable approach: Comparative toxicology of selective vs. non‐selective vinyl sulfone analogues, alongside reversible inhibitors matched for exposure, could disentangle these mechanisms.
- What degree of cruzain isoform engagement is required for parasitological cure?
  Most CZ inhibitor programmes implicitly assumed that broad inhibition across CZ isoforms is desirable. However, isoform expression and functional relevance may differ between parasite stages, particularly in chronic intracellular infection.
  ○ Translational implication: Over‐inhibition of non‐essential isoforms may unnecessarily compress selectivity windows and exacerbate off‐target liabilities
  ○ Experimentally testable approach: Isoform‐resolved biochemical assays combined with stage‐specific infection models could define minimal target engagement profiles compatible with parasitological clearance.
- Can tunable electrophiles balance potency and metabolic stability?
  The metabolic instability observed for thiosemicarbazones illustrates the risks associated with highly reactive electrophiles. Emerging strategies employing reversible covalent or attenuated electrophilic warheads may offer a compromise between sustained target engagement and acceptable metabolic persistence.
  ○ Translational implication: Warhead choice should be treated as a central optimisation variable rather than a fixed scaffold property.
  ○ Experimentally testable approach: Systematic comparison of irreversible, reversible covalent, and non‐covalent inhibitors within matched scaffolds under identical exposure conditions.
- Can multiparametric computational pipelines outperform affinity‐centric ones in prospective hit triage?
  Despite increasing methodological sophistication, computational approaches in CZ inhibitor discovery have not altered translational outcomes. This raises the possibility that failure arises not from algorithmic limitations but from objective misspecification.
  ○ Translational implication: Affinity‐centric computational optimisation may be intrinsically misaligned with the requirements of intracellular drug exposure and durability.
  ○ Experimentally testable approach: Parallel machine‐learning models trained on enzymatic potency alone versus multiparametric endpoints could be benchmarked for prospective prediction of translationally viable candidates.

Collectively, these open questions operationalise the MPO framework and transform retrospective diagnosis into a forward‐looking translational agenda. Each question addresses a specific failure mode identified in earlier sections and is experimentally tractable using existing methodologies. Progress along any single axis would meaningfully advance the field; progress across several would redefine it. In the concluding section, we integrate these implications to articulate how CZ inhibitor discovery can move beyond historical optimisation traps towards clinically relevant outcomes.

## 7 Conclusion and Perspectives

Three decades and 215 potent inhibitors have produced no clinical candidate for CZ; an outcome that is difficult to attribute primarily to deficiencies in target biology, structural knowledge or computational sophistication, but instead reflects a systematic misalignment between optimisation strategies and the biological determinants of efficacy in intracellular parasitic infection. The central contribution of this review is to reformulate this problem within an MPO framework that treats potency, selectivity, physicochemical properties governing intracellular accumulation, and metabolic stability as simultaneous design constraints rather than sequential milestones. This framework does not require new experimental technologies; it requires a reordering of priorities and explicit recognition that early trade‐offs determine late‐stage viability.

The implications extend beyond CZ. Intracellular targets housed within compartmentalised and pH‐heterogeneous environments, whether in other parasitic diseases, lysosomal storage disorders, or oncology, appear to face similar optimisation constraints. The recurring failure to translate high‐affinity inhibitors into clinical agents across these domains suggests that the lessons distilled here may generalise to other structurally tractable intracellular enzyme targets, although the weight of CZ‐specific aggravating factors (Section 5.4) means that any such generalisation requires careful case‐by‐case calibration.

Viewed through this lens, the absence of a CZ clinical candidate is not evidence that the target is untenable. It is evidence that the field has been optimising against an incomplete objective function. The framework presented here is immediately applicable: the 215 potency‐qualified inhibitors already in ChEMBL3563 constitute an underutilised dataset for prospective MPO‐guided rescreening, requiring no new chemistry but only a respecified objective function. We hypothesise that systematic application of the MPO criteria outlined in Table 3 to this dataset would identify a feasibility‐envelope‐compliant subset on the order of 10–50 compounds. This estimate is consistent with the empirical observation that compounds achieving favourable enzymatic‐to‐cellular translation (e.g., the cyclic imide compound 59 of Ferreira et al. 2019; the thiosulfonate TSO‐3 of Cerutti et al. 2025; the quinazoline 1s of Silva et al. 2021) currently number only in the single digits across the published literature. This prediction is directly testable by retrospective rescreening and, if confirmed, would identify the true starting point for prospective translation. Whether this strategic reorientation occurs will determine whether CZ inhibitor discovery transitions from a paradigm of incremental potency gains towards the systems‐level, exposure‐aware optimisation that intracellular parasitic targets demonstrably require.

## Author Contributions

**Bruna Costa Zorzanelli:** formal analysis, data curation, writing – original draft, writing – review and editing. **Tácio Vinício Amorim Fernandes:** conceptualization, investigation, funding acquisition, writing – original draft, methodology, validation, visualization, writing – review and editing, formal analysis, project administration, data curation, supervision, resources. **Guilber Valério Chaves Moura de Souza:** writing – original draft, data curation. **Lidia Corrêa Parra:** writing – review and editing, formal analysis, data curation, writing – original draft. **Caroline Rodrigues Chaves dos Reis:** writing – original draft, writing – review and editing, formal analysis, data curation, supervision, methodology, validation, visualization. **Nubia Boechat:** conceptualization, investigation, funding acquisition, writing – original draft, formal analysis, supervision, project administration, writing – review and editing, resources. **Hellen Valério Chaves Moura de Souza:** writing – original draft, writing – review and editing, formal analysis, data curation, methodology, validation, visualization. **Lucas Villas Bôas Hoelz:** conceptualization, investigation, writing – original draft, writing – review and editing, visualization, validation, methodology, formal analysis, supervision, data curation.

## Funding

We thank the National Council for Scientific and Technological Development (CNPq 447623/2024‐9 and 440006/2022‐8) and the Foundation for Research Support of the State of Rio de Janeiro (FAPERJ‐JCNE E‐26/201.268/2021) for all support. This work was supported in part by the Coordenação de Aperfeiçoamento de Pessoal de Nível Superior—Brasil (CAPES)—Finance Code 001.

## Consent

The authors have nothing to report.

## Conflicts of Interest

The authors declare no conflicts of interest.

## Supporting information


**Figure S1:** Distribution of pChEMBL values for cruzipain inhibitors (CHEMBL3563).
**Figure S2:** Life cycle of *Trypanosoma cruzi*.
**Figure S3:** Structural architecture and subsites of cruzain (PDB ID: 3KKU).
**Figure S4:** Detailed catalytic mechanism of cruzain.
**Figure S5:** Ligand efficiency analysis of the curated dataset.
**Table S1:** Enzymatic inhibition and translational outcomes of cruzain inhibitor classes.

## Data Availability

The bioactivity data analysed in this review were retrieved from the publicly accessible ChEMBL database (target CHEMBL3563, cruzipain; https://www.ebi.ac.uk/chembl/explore/target/CHEMBL3563, accessed February 6, 2026). The curation workflow and filtering criteria used to generate the compound statistics and pChEMBL distributions reported herein are described in the Supporting Information. All other data discussed in this review are derived from previously published studies cited in the reference list. No new experimental data were generated by the authors. The curated dataset and the Python scripts used for the ligand‐efficiency analysis are available from the corresponding author upon reasonable request.
