# Supplementary material for: Cruzain Inhibitors for Chagas Disease: Anticorrelated Optimisation Landscapes and the Multiparametric Path to Clinical Candidates
Source: Chem Biol Drug Des. 2026 Jun 14;107(6):e70340. doi: 10.1111/cbdd.70340 (PMC13266285; doi:10.1111/cbdd.70340)
Supplement: Supplementary file 1 — Figure S1: Distribution of pChEMBL values for cruzipain inhibitors (CHEMBL3563). Figure S2: Life cycle of Trypanosoma cruzi. Figure S3: Structural architecture and subsites of cruzain (PDB ID: 3KKU). Figure S4: Detailed catalytic mechanism of cruzain. Figure S5: Ligand efficiency analysis of the curated dataset. Table S1: Enzymatic inhibition and translational outcomes of cruzain inhibitor classes. [file CBDD-107-e70340-s001.docx]

**SUPPLEMENTARY MATERIAL**

**Cruzain Inhibitors for Chagas Disease: Anticorrelated Optimisation Landscapes and the Multiparametric Path to Clinical Candidates**

Caroline Rodrigues Chaves dos Reis (<https://orcid.org/0000-0001-8982-242X>)^1,2^,

Hellen Valério Chaves Moura de Souza (<https://orcid.org/0000-0003-0152-0545>)^1,2^,

Lidia Corrêa Parra (<https://orcid.org/0009-0005-0134-4547>)^1,2^,

Guilber Valério Chaves Moura de Souza (<https://orcid.org/0009-0009-2689-3488>)^2,3^,

Bruna Costa Zorzanelli (<https://orcid.org/0000-0002-5173-4624>)^4,5^,

Nubia Boechat (<https://orcid.org/0000-0003-0146-2218>)^1,2^,

Lucas Villas Bôas Hoelz (<https://orcid.org/0000-0001-5592-3089>)^1,6^,

Tácio Vinício Amorim Fernandes (<https://orcid.org/0000-0001-5881-9519>)^1,2,*^

^1^Fundação Oswaldo Cruz, Fiocruz, Instituto de Tecnologia em Fármacos, Programa de Pós-Graduação em Pesquisa Translacional em Fármacos e Medicamentos, Rio de Janeiro, RJ, Brazil.

^2^Fundação Oswaldo Cruz, Fiocruz, Instituto de Tecnologia em Fármacos, Laboratório de Síntese de Fármacos (LASFAR), Rio de Janeiro, RJ, Brazil.

^3^Universidade Federal Fluminense (UFF), Campus Praia Vermelha, Niterói, RJ, Brazil.

^4^Universidade Estácio de Sá, Campus Niterói, Niterói, RJ, Brazil.

^5^Centro Universitário Serra dos Órgãos (UNIFESO), Teresópolis, RJ, Brazil.

^6^Instituto Federal do Rio de Janeiro (IFRJ), Laboratório Computacional de Química Medicinal, Pinheiral, RJ, Brazil.

*Correspondence details: Tácio Vinício Amorim Fernandes ([tacio.fernandes@fiocruz.br](mailto:tacio.fernandes@fiocruz.br)), Fundação Oswaldo Cruz, Instituto de Tecnologia em Fármacos, Farmanguinhos, Laboratório de Síntese Fármacos - LASFAR, Rio de Janeiro, RJ, Brazil.

## **Data Retrieval and Curation of Cruzipain Inhibition Data**

Bioactivity data were retrieved from the ChEMBL database for target CHEMBL3563, corresponding to cruzipain (also annotated as cruzain or major cysteine proteinase), a single-protein target from Trypanosoma cruzi. The dataset was downloaded in CSV format directly from the ChEMBL web interface using the predefined filter “Bioactivity data for target CHEMBL3563 (Cruzipain) – IC_50_” (accessed February 6, 2026). The initial dataset comprised 1048 activity records corresponding to 765 unique Molecule ChEMBL IDs.

Only entries reporting Standard Type = IC_50_ and containing a numeric pChEMBL value were retained, yielding 668 IC_50_ records suitable for quantitative analysis. In ChEMBL, the pChEMBL value is defined as -log10 of the standardized molar potency; therefore, for IC_50_ endpoints, pChEMBL is equivalent to pIC_50_. A potency threshold of pChEMBL ≥ 6 was applied, corresponding to IC_50_ ≤ 1 µM on the molar scale. This filtering step resulted in 317 potency-qualified IC_50_ records.

When multiple activity entries were available for the same compound, records were deduplicated by Molecule ChEMBL ID, retaining the most potent measurement (highest pChEMBL value); in cases of identical pChEMBL values, the entry with the lowest IC_50_ value (after unit standardization) was retained. This procedure yielded a final curated dataset comprising 215 unique cruzipain inhibitors meeting the predefined potency criterion.

Reported IC_50_ values were converted to micromolar (µM) units when necessary to ensure consistency across measurements. Activity relations indicating censored values (e.g., “>” or “>>”) were excluded from quantitative distribution analyses to avoid bias introduced by left-censored potency values. The curated dataset was subsequently used to compute descriptive statistics and to generate distributions of pChEMBL values, IC_50_ values, and log-transformed IC_50_ values (log10 IC_50_ [µM]). The distribution of pChEMBL values before and after application of the potency threshold is shown in Figure S1. All data processing and statistical analyses were performed using Python libraries.

## **Supplementary Figure S1. Distribution of pChEMBL values for cruzipain inhibitors (CHEMBL3563).**


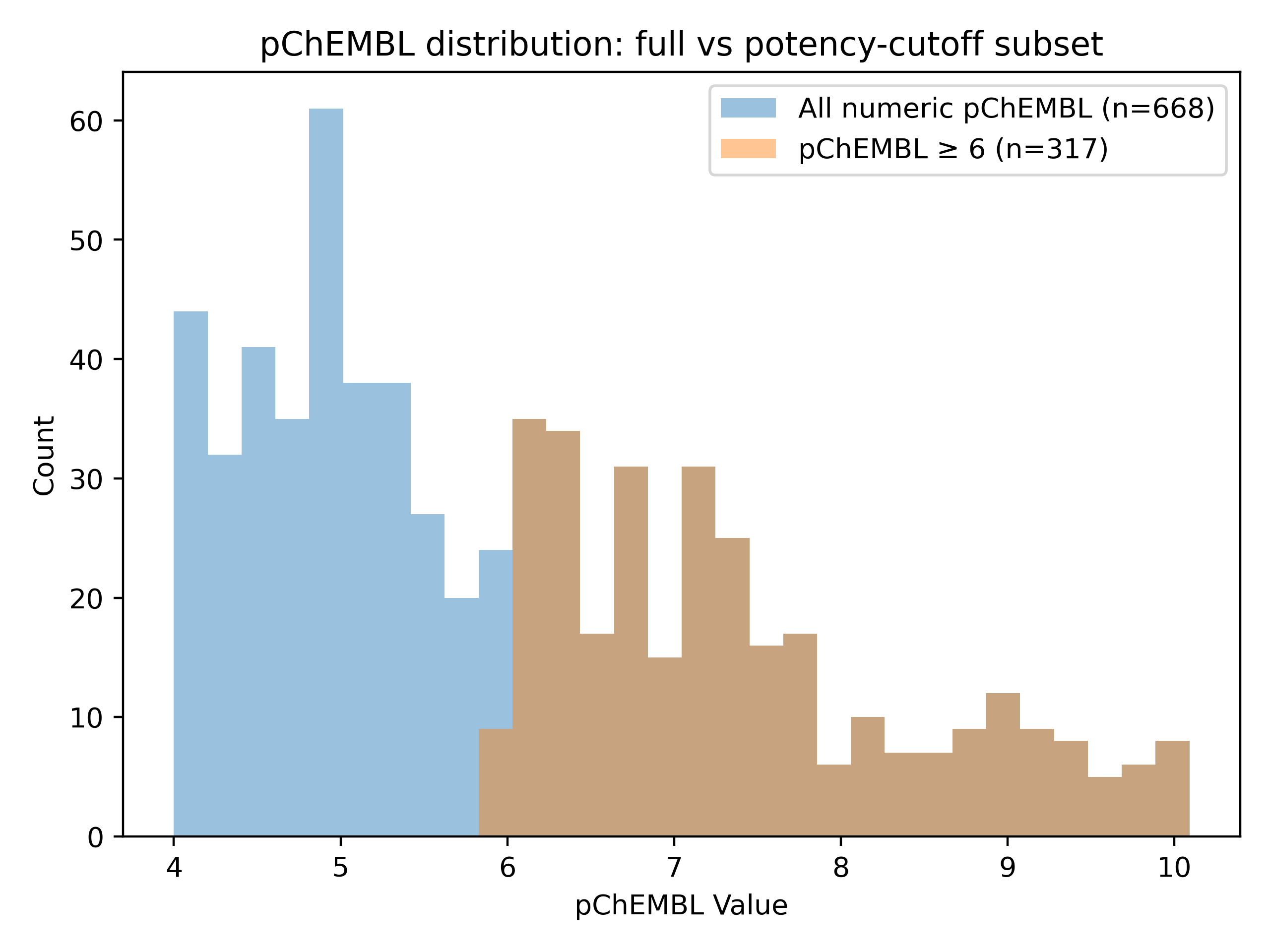


**Supplementary Figure S1.** Overlay histogram showing the distribution of all IC_50_ records with numeric pChEMBL values (n = 668) and the subset meeting the predefined potency criterion (pChEMBL ≥ 6; n = 317 or 215 unique inhibitors qualified after deduplication by Molecule ChEMBL ID). The cutoff corresponds to IC_50_ ≤ 1 µM on the molar scale. Counts are shown per bin; histograms share identical binning to enable direct comparison of potency enrichment following threshold application.

## **Supplementary Figure S2. Life cycle of *Trypanosoma cruzi***


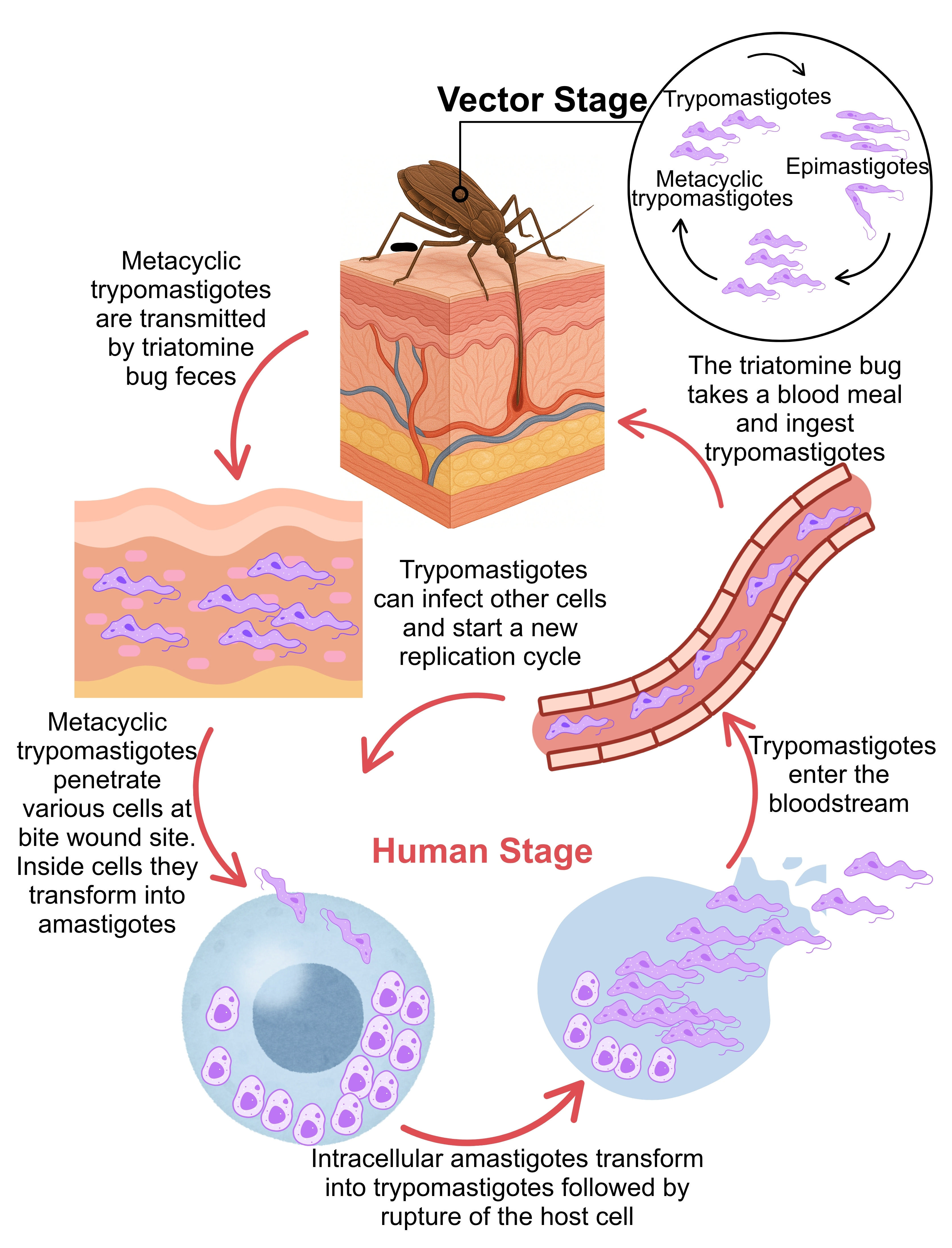


**Supplementary Figure S2.** Schematic representation of the major developmental stages of *T. cruzi*, including epimastigotes in the insect vector, bloodstream trypomastigotes, and intracellular amastigotes in mammalian host tissues. Cruzain (CZ) is expressed and functionally active across all life-cycle stages; however, from a therapeutic perspective, disease persistence and pathology during chronic infection are driven predominantly by intracellular amastigotes. This figure provides biological context for the emphasis on intracellular exposure, compartmentalisation, and pH-dependent activity discussed in the main text.

**Purpose in the Supplementary**

This figure provides biological background supporting the translational focus of the review, without repeating material central to the argumentation in the main manuscript.

## **Supplementary Figure S3. Structural Architecture and Subsites of Cruzain (PDB ID: 3KKU)**

Overall bilobal architecture of cruzain (CZ), highlighting the catalytic triad and canonical substrate-binding subsites. Figure S3 provides structural context for the selectivity constraints discussed in the main text. By mapping the catalytic triad and the S1-S3/S1’ subsites, it illustrates why CZ is highly tractable for structure-based design while also explaining why selectivity over host cathepsins must be engineered through limited peripheral subsite differences rather than through the conserved catalytic core.


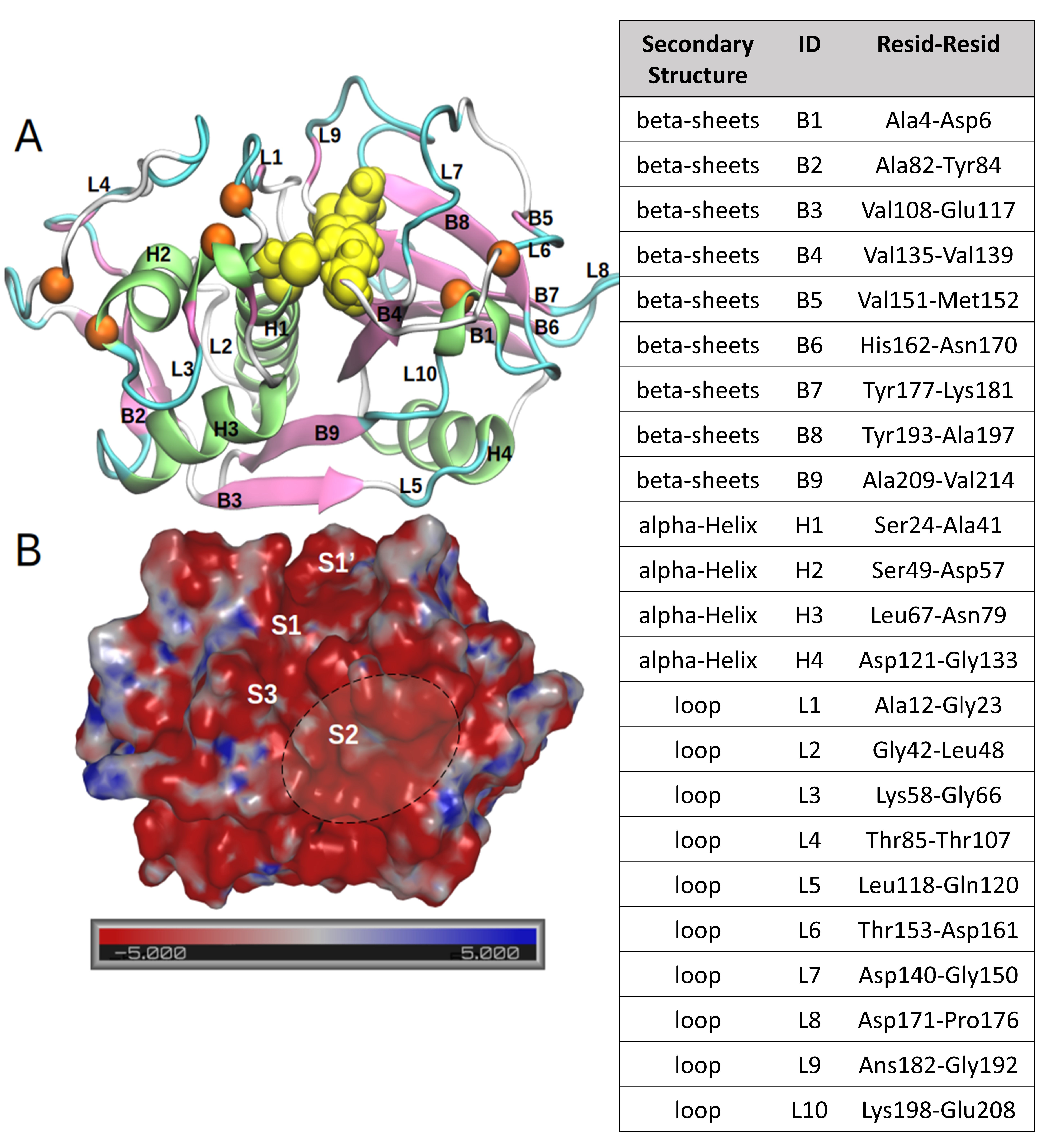


**Supplementary Figure S3.** Structural Architecture and Subsites of Cruzain (PDB ID: 3KKU). New cartoon representation of mature cruzain (215 residues), highlighting the structural features that underlie both its exceptional tractability for structure-based drug design and the intrinsic constraints on selectivity optimisation. (A) bilobal architecture with α-helical L-domain (lime; helices H1-H4, residues Ser24- Gly133) and β-barrel R-domain (mauve; strands B1-B9, residues Ala4-Val214); catalytic triad residues Cys25, His162, Asn182 (yellow spheres; 1.5 Å van der Waals radius) positioned at domain interface; three disulfide bridges stabilizing domain architecture: Cys22-Cys63, Cys56-Cys101, Cys155-Cys203 (orange spheres). (B) substrate-binding subsites S1-S3 and S1` (transparent molecular surface, coloured by electrostatic potential: blue +5 kT/e, red -5 kT/e); The S2 subsite (dominant specificity determinant, dashed circle) comprises Leu67, Met68, Ala138, Leu157, Leu160, and Glu208. Inset table: secondary structure elements with residue ranges (H, α-helix; B, β-strand; L, loop). Structural coordinates obtained from PDB entry 3KKU (resolution = 1.28 Å, R-factor = 0.1162) (Ferreira et al., 2010). Visualization generated with PyMOL 2.5 (Schrödinger LLC).

## **Supplementary Figure S4. Detailed catalytic mechanism of cruzain**

The cruzain proteolytic cycle depicted below is inferred from high-resolution crystallographic analysis of the native enzyme (McGrath et al., 1995) combined with the mechanistic framework established for papain-family cysteine proteases (Lecaille et al., 2002; Turk et al., 2012). Quantum mechanics/molecular mechanics (QM/MM) studies of cruzain (CZ) have not reconstructed the complete hydrolytic cycle with a natural peptide substrate; instead, they have characterised the energetics and electronic structure of the Cys25-His162 ion pair during covalent engagement with diverse inhibitor warheads, including dipeptidyl nitriles (dos Santos et al., 2018; Silva et al., 2020), peptidyl-2,3-epoxyketones (Arafet et al., 2017), dipeptidyl nitroalkenes across CZ, rhodesain, and cathepsin L (Arafet et al., 2020), and aryl-thiosemicarbazones (Martins et al., 2023). Collectively, these investigations validate the representation of the reactive thiolate-imidazolium ion pair and its engagement of electrophilic carbons through concerted proton transfer and nucleophilic attack, as depicted in steps 2-4. No current QM/MM study directly compares activation free energies between neutral and lysosomal pH regimes for the natural catalytic cycle of CZ; the pH-dependent considerations developed in Figure 3 of the main text should therefore be understood as a qualitative expectation consistent with the pKa environment of the triad, not as a quantitative QM/MM-derived prediction.


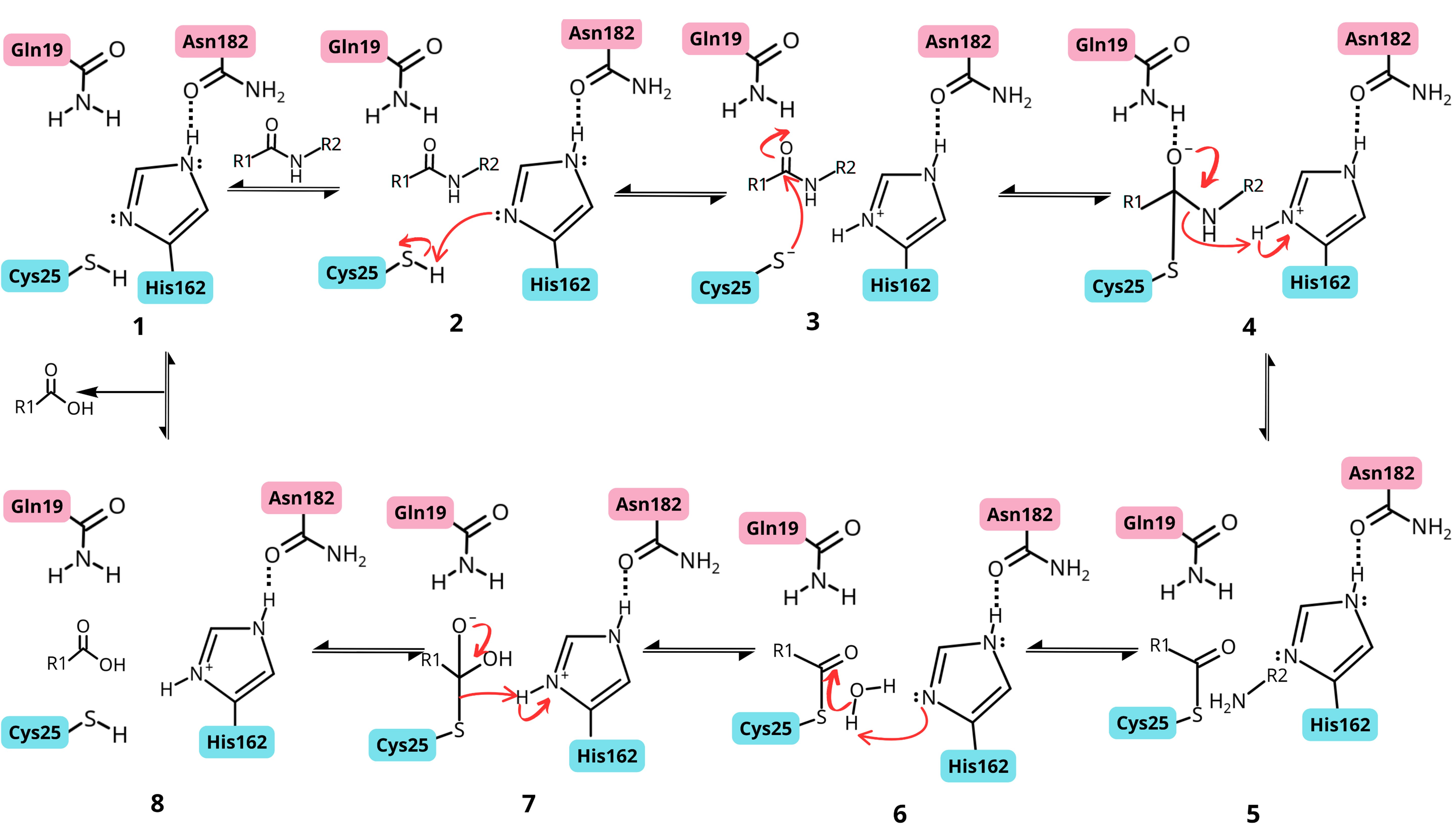


**Supplementary Figure S4. Stepwise catalytic mechanism of cruzain (CZ) cysteine protease.** Detailed representation of the CZ catalytic cycle, adapted from experimental and QM/MM studies. The mechanism proceeds through sequential acylation and deacylation steps involving the Cys25-His162-Asn182 catalytic triad, with stabilisation of tetrahedral intermediates by the oxyanion hole. Proton-transfer events and intermediate states are shown to provide mechanistic background for the pH-dependent catalytic states summarised in Figure 3 of the main text. This figure is provided for mechanistic completeness and is not required for interpretation of the primary translational conclusions.

**Purpose in the Supplementary**

This figure preserves mechanistic detail for specialist readers while avoiding excessive biochemical detail in the main text.

## **Supplementary Figure S5. Ligand Efficiency Analysis of the Curated Dataset**

To test whether potency gains across the curated CHEMBL3563 dataset have been driven by increases in molecular size rather than by improvements in per-heavy-atom binding efficiency (the molecular obesity pattern described in the broader anti-infective literature (Leeson and Springthorpe, 2007; Hopkins et al., 2014)) ligand efficiency (LE) was computed for each of the 215 curated inhibitors using the standard formulation

$$LE=\frac{1.37\times{pIC}_{50}}{N_{heavy}}$$

where the prefactor 1.37 corresponds to RT ln(10) at 298.15 K, expressing LE as a binding free-energy proxy in units of kcal/mol per heavy atom. Heavy-atom counts (*N_heavy_*) were derived from the canonical SMILES strings using RDKit (release 2024.09); molecular weights were taken directly from the ChEMBL record.

Pearson and Spearman correlations between potency (pIC_50_) and the two structural descriptors – molecular weight (MW) and ligand efficiency (LE) – were computed across the entire curated set (n = 215). Across all four coefficients, potency was found to track molecular size substantially more strongly than per-atom efficiency: Pearson r(MW, pIC_50_) = +0.610 and Spearman *ρ* = +0.519 (both *p* < 1 x 10^-4^), against r(LE, pIC_50_) = ‑0.213 (*p* = 1.6 x 10^-3^) and *ρ* = ‑0.188 (*p* = 5.7 x 10^-3^). The weak LE-potency association is mechanistically informative: as compounds become more potent across the curated set, the average free-energy contribution per heavy atom decreases, indicating that potency gains have not been accompanied by proportionate gains in binding economy.

Stratifying the dataset into potency tertiles further resolves where this pattern emerges. The mid-potency tertile (T2; pIC_50_ ≈ 7.4) achieves the highest mean ligand efficiency (LE ≈ 0.44 kcal/mol/atom) at a MW comparable to the low-potency tertile (T1; MW ≈ 385‑395 Da), consistent with genuine interaction optimisation. The transition from T2 to the high-potency tertile (T3; pIC_50_ ≈ 9.1) is achieved by adding, on average, ~18 heavy atoms (MW increasing from ≈395 Da to ≈633 Da; +60%) while LE simultaneously falls to 0.31 kcal/mol/atom⁻¹, a regime in which incremental potency is purchased through molecular growth rather than through more efficient engagement of the CZ active site. This pattern is consistent with retrospective trends reported across other anti-infective and protease-inhibitor classes (Leeson and Springthorpe, 2007; Hopkins et al., 2014) and supports, on the curated CZ dataset specifically, the empirical interpretation of the potency-exposure anticorrelation discussed in Section 5.5 of the main text.

The two correlations are visualised in Supplementary Figure S5. The complete analysis pipeline (from raw ChEMBL records to LE computation, correlations and tertile stratification) is reproducible from the curated dataset (*file curated_dataset.csv*) using the accompanying Python script (*le_analysis.py*); both are available from the corresponding author upon reasonable request.

Supplementary Figure S5. Ligand efficiency vs. molecular weight in the curated cruzipain inhibitor dataset.


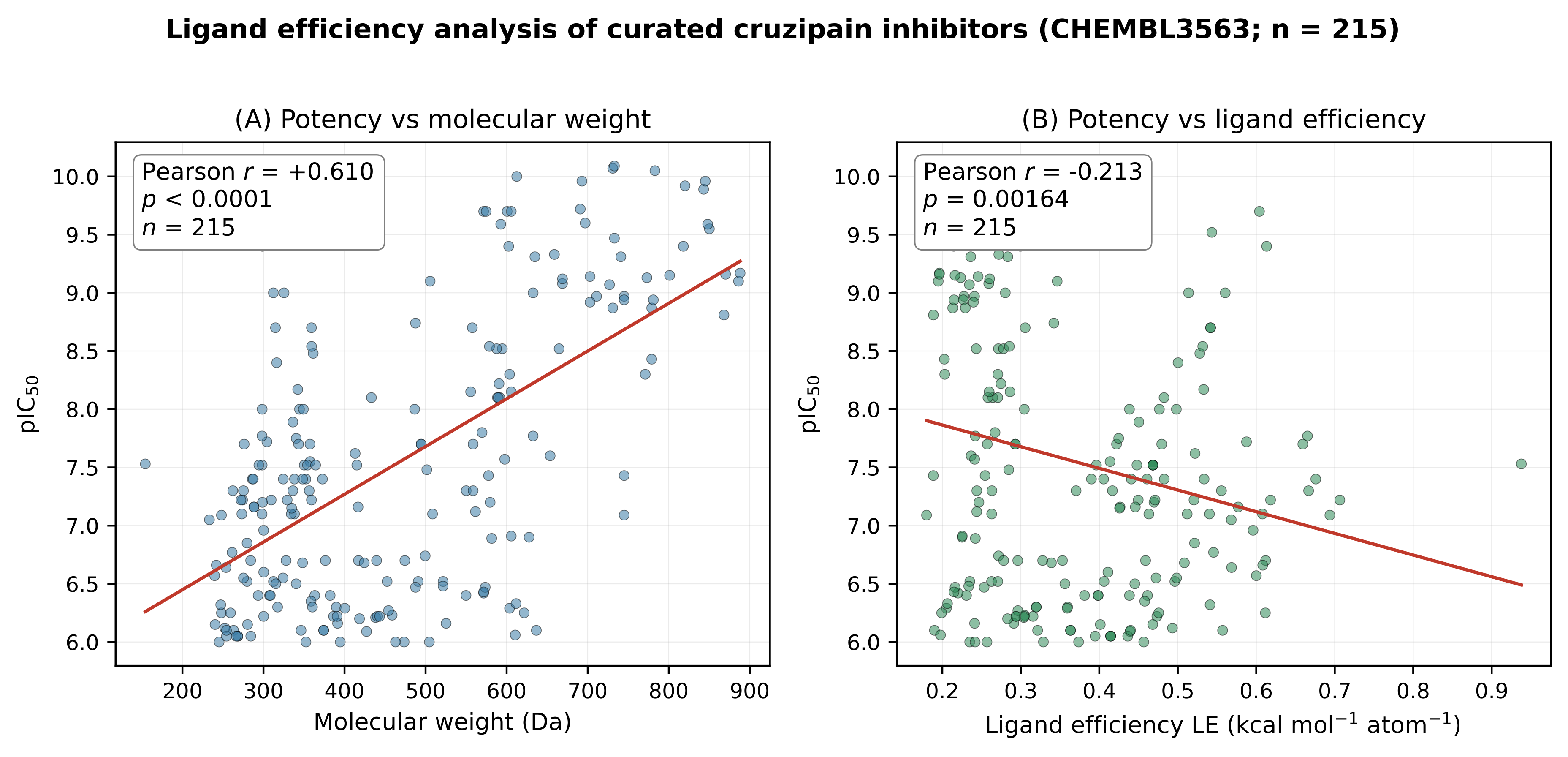


**Supplementary Figure S5. Ligand efficiency vs. molecular weight.** Two-panel scatter analysis of n = 215 unique cruzipain inhibitors curated from ChEMBL target CHEMBL3563 (pChEMBL ≥ 6, deduplicated by Molecule ChEMBL ID; see preceding Supplementary Methods). (A) Potency (pIC_50_) plotted against molecular weight (Da). The least-squares regression line (red) and Pearson statistics shown in the inset confirm a strong positive association between molecular size and biochemical potency. (B) Potency (pIC_50_) plotted against ligand efficiency (LE = 1.37 x pIC_50_/N_heavy atoms_; kcal/mol per heavy atom). The weak Pearson correlation (r = ‑0.213, p = 1.6 x 10^-3^) indicates that compounds with higher potency tend to display lower per-heavy-atom binding efficiency, consistent with the molecular obesity pattern in which potency gains are achieved primarily through molecular growth rather than through improvements in interaction efficiency. The disparity in correlation magnitude (|r(MW, pIC50)| = 0.610 vs. |r(LE, pIC50)| = 0.213) provides quantitative support, on the CZ dataset specifically, for the empirical interpretation of the potency-exposure anticorrelation developed in Section 5.5 of the main text.

## **Supplementary Table S1. Enzymatic inhibition and translational outcomes of cruzain inhibitor classes**

**Supplementary Table S1. Representative Examples of Enzymatic-to-Cellular Potency Disconnects in Cruzain Inhibitor Classes**

| **Compound class** | **Representative example** | **Cruzain IC_50_ (enzyme)** | **Cellular EC_50_ (*T. cruzi* amastigotes)** | **Approx. fold disconnect^†^** | **Ref.** | **Notes** |
| --- | --- | --- | --- | --- | --- | --- |
| **Benzimidazoles** | 1,2,5-Trisubstituted benzimidazole derivatives | 5-50 μM (best compounds IC_50_ = 5-16 μM) | Typically >100 μM (weak activity) | ~6-20-fold | Caputto et al. (2012) | Poor cellular translation attributed to limited permeability; *T. cruz*i growth inhibition IC_50_= 6-16 μM for epimastigotes but substantially weaker activity against intracellular amastigotes |
| **Thiosemicarbazones** | 5,6-Dimethoxyindan-1-one N-(4-chlorophenyl) thiosemicarbazone | Best compounds IC_50_ ~380 nM (reported for series); 67% inhibition at 100 μM for representative analogues | Anti-*T. cruzi* activity in low μM range | ~5-10x (estimated) | Caputto et al. (2012); Du et al. (2002) | Weak correlation between cruzain inhibition and trypanocidal activity; enzymatic potency does not translate proportionally to cellular efficacy |
| **Cyclic imides** | Compound 59 | 0.6 μM | 1.0 μM | ~1.7x (favourable) | Ferreira et al. (2019) | One of the few reported examples showing reasonable enzymatic-to-cellular translation, demonstrating that the disconnect is surmountable through rational optimisation of permeability |

^†^ Fold disconnect defined as (cellular EC_50_)/(enzymatic IC_50_). Values are approximate when enzymatic IC_50_ or cellular EC_50_ were not explicitly reported for the same compound and are intended to illustrate qualitative trends rather than precise quantitative ratios.

**Key:**

- Fold Disconnect = (Cellular EC_50_) / (Enzymatic IC_50_).
- Benzimidazole data compiled from references (Caputto et al., 2012) and (Ríos et al., 2013).
- Thiosemicarbazone data primarily from (Du et al., 2002) showing best enzymatic potency IC_50_ = 380 nM but cellular activity only at μM concentrations.
- The cyclic imide example (Compound 59) serves as a positive control, demonstrating successful translation when permeability is optimised.

# **References**

**Arafet, K.; Ferrer, S.; González, F. V.; Moliner, V.** Quantum mechanics/molecular mechanics studies of the mechanism of cysteine protease inhibition by peptidyl-2,3-epoxyketones. **Physical Chemistry Chemical Physics**, v. 19, n. 20, p. 12740-12748, 2017. DOI: 10.1039/c7cp01726j

**Arafet, K.; González, F. V.; Moliner, V.** Quantum Mechanics/Molecular Mechanics Studies of the Mechanism of Cysteine Proteases Inhibition by Dipeptidyl Nitroalkenes. **Chemistry - A European Journal**, v. 26, n. 9, p. 2002-2012, 2020. DOI: 10.1002/chem.201904513

Caputto, M. E.; Ciccarelli, A.; Frank, F.; Moglioni, A. G.; Moltrasio, G. Y.; Vega, D.; Lombardo, E.; Finkielsztein, L. M. Synthesis and biological evaluation of some novel 1-indanone thiazolylhydrazone derivatives as anti-*Trypanosoma cruzi* agents. **European Journal of Medicinal Chemistry**, v. 55, p. 155-163, 2012. DOI:10.1016/j.ejmech.2012.07.013

**Dos Santos, A. M.; Cianni, L.; De Vita, D.; Rosini, F.; Leitão, A.; Laughton, C. A.; Lameira, J.; Montanari, C. A.** Experimental study and computational modelling of cruzain cysteine protease inhibition by dipeptidyl nitriles. **Physical Chemistry Chemical Physics**, v. 20, n. 37, p. 24317-24328, 2018. DOI: 10.1039/c8cp03320j

Du, X.; Guo, C.; Hansell, E.; Doyle, P. S.; Caffrey, C. R.; Holler, T. P.; McKerrow, J. H.; Cohen, F. E. Synthesis and structure-activity relationship study of potent trypanocidal Thio Semicarbazone inhibitors of the Trypanosomal cysteine protease cruzain. **Journal of Medicinal Chemistry**, v. 45, p. 2695-2707, 2002. DOI:10.1021/jm010459j

Ferreira, R. A. A.; Pauli, I.; Sampaio, T. S.; De Souza, M. L.; Ferreira, L. L. G.; Magalhães, L. G.; Rezende, C. O. Jr.; Ferreira, R. S.; Krogh, R.; Dias, L. C.; Andricopulo, A. D. Structure-Based and Molecular Modeling Studies for the Discovery of Cyclic Imides as Reversible Cruzain Inhibitors with Potent Anti-Trypanosoma cruzi Activity. **Frontiers in Chemistry**, v. 7, p. 798, 2019. DOI: 10.3389/fchem.2019.00798

Ferreira, R. S.; Simeonov, Anton.; Jadhav, A.; Eidam O.; Mott, B. T.; Keiser, M. J.; McKerrow, J. H.; Maloney, D. J.; Irwin, J. J.; Shoichet, B. K.. Complementarity Between a Docking and a High-Throughput Screen in Discovering New Cruzain Inhibitors. **Journal of Medicinal Chemistry** **2010** 53 (13), 4891-4905. DOI: 10.1021/jm100488w

Hopkins, A. L.; Keserü, G. M.; Leeson, P. D.; Rees, D. C.; Reynolds, C. H. The role of ligand efficiency metrics in drug discovery. **Nature Reviews Drug Discovery**, v. 13, p. 105-121, 2014. DOI:10.1038/nrd4163

Leeson, P. D.; Springthorpe, B. The influence of drug-like concepts on decision-making in medicinal chemistry. **Nature Reviews Drug Discovery**, v. 6, n. 11, p. 881–890, 2007. DOI:10.1038/nrd2445

**Silva, J. R. A.; Cianni, L.; Araujo, D.; Batista, P. H. J.; de Vita, D.; Rosini, F.; Leitão, A.; Lameira, J.; Montanari, C. A.** Assessment of the Cruzain Cysteine Protease Reversible and Irreversible Covalent Inhibition Mechanism. **Journal of Chemical Information and Modeling**, v. 60, n. 3, p. 1666-1677, 2020. DOI: 10.1021/acs.jcim.9b01138

Ríos, N.; Varela, J.; Birriel, E.; González, M.; Cerecetto, H.; Merlino, A.; Porcal W. Trypanosomatid Disease Drug Discovery and Target Identification. **Future Medicinal Chemistry** 5:15, pages 1719-1732, 2013. DOI: 10.4155/fmc.13.160
